# Distinct functions of dimeric and monomeric scaffold protein Alix in regulating F-actin assembly and loading of exosomal cargo

**DOI:** 10.1016/j.jbc.2022.102425

**Published:** 2022-08-27

**Authors:** Xiaohui Qiu, Yvan Campos, Diantha van de Vlekkert, Elida Gomero, Ajay C. Tanwar, Ravi Kalathur, Jason A. Weesner, Antonella Bongiovanni, Jeroen Demmers, Alessandra d’Azzo

**Affiliations:** 1 Department of Genetics, St Jude Children’s Research Hospital, Memphis, Tennessee, USA; 2 Department of Structural Biology, St Jude Children’s Research Hospital, Memphis, Tennessee, USA; 3 Department of Anatomy and Neurobiology, College of Graduate Health Sciences, University of Tennessee Health Science Center, Memphis, Tennessee, USA; 4 Institute of Biomedical Research and Innovation (IRIB), National Research Council (CNR) of Italy, Palermo, Italy; 5 Proteomics Center, Erasmus University Medical Center, Rotterdam, the Netherlands

**Keywords:** alix, syntenin, exosome, disulfide, dimerization, actin, cytoskeleton, cDNA, complementary DNA, MEF, mouse embryonic fibroblast, MSCV, murine stem cell virus, MVB, multivesicular body, PRD, proline-rich domain, SEC, size-exclusion chromatography, TBS, tris-buffered saline

## Abstract

Alix is a ubiquitously expressed scaffold protein that participates in numerous cellular processes related to the remodeling/repair of membranes and the actin cytoskeleton. Alix exists in monomeric and dimeric/multimeric configurations, but how dimer formation occurs and what role the dimer has in Alix-mediated processes are still largely elusive. Here, we reveal a mechanism for Alix homodimerization mediated by disulfide bonds under physiological conditions and demonstrate that the Alix dimer is enriched in exosomes and F-actin cytoskeleton subcellular fractions. Proteomic analysis of exosomes derived from *Alix*^*−/−*^ primary cells underlined the indispensable role of Alix in loading syntenin into exosomes, thereby regulating the cellular levels of this protein. Using a set of deletion mutants, we define the function of Alix Bro1 domain, which is solely required for its exosomal localization, and that of the V domain, which is needed for recruiting syntenin into exosomes. We reveal an essential role for Cys^814^ within the disordered proline-rich domain for Alix dimerization. By mutating this residue, we show that Alix remains exclusively monomeric and, in this configuration, is effective in loading syntenin into exosomes. In contrast, loss of dimerization affects the ability of Alix to associate with F-actin, thereby compromising Alix-mediated cytoskeleton remodeling. We propose that dimeric and monomeric forms of Alix selectively execute two of the protein’s main functions: exosomal cargo loading and cytoskeleton remodeling.

Alix, also known as Pdcd6ip or AIP1 (apoptosis-linked gene-2 (ALG-2)-interacting protein 1), is a multivalent scaffold protein, whose primary structure embeds three major modules: the N-terminal Bro1 domain, the central V-shaped domain, and the C-terminal proline-rich domain or PRD. Several structural studies of the Bro1 and V domains have revealed a characteristic boomerang shape of the protein (1, 2, 3). However, relatively little is known about the C-terminal PRD, which is highly disordered. PRDs are among the most prevalent modules in eukaryotic proteins (4), although their structural characteristics have been hard to define due to the challenge posed by their intrinsically disordered nature, which makes their expression *in vitro* difficult to achieve (1, 5).

The Bro1, V, and PRD modules of Alix each contains binding sites for diverse cellular components that are themselves parts of large membrane complexes or the actin cytoskeleton (6, 7, 8). Some of Alix protein partners that interact with its Bro1 domain include F-actin, the ESCRTIII component, CHMP4 (charged multivesicular body [MVB] protein 4), and the lipid bis[monoacylglycero]phosphate (BMP) (also termed lysobisphosphatidic acid [LBPA]) (9, 10, 11). The interacting partners binding to the V module include cortactin, ubiquitin, activated EGFR (epidermal growth factor receptor), HIV P6 or EIAV p9, and syntenin (1, 9, 12, 13, 14). Although the structure of the C-terminal PRD remains unsolved, it was shown to bind to F-actin, ALG-2, the ESCRTI component TSG101 (tumor-susceptibility gene 101), midbody protein CEP55 (Centrosomal protein of 55 kDa), endocytic adapter protein CIN85 (Cbl-interacting protein of 85 kDa, also known as SETA), and endocytic protein endophilin (9, 15, 16, 17, 18, 19).

Inferred from these interactions, Alix has been largely implicated in two main functions: first, by interacting with components of the ESCRT machineries, Alix recruits ESCRTIII and takes part in endocytosis and recycling of membrane receptors, MVB and exosome biogenesis, viral budding, cytokinesis, and cell membrane repair (11, 13, 16, 20, 21, 22, 23, 24, 25). Secondly, Alix participates in actin cytoskeleton remodeling by binding to F-actin and F-actin–interacting proteins (6, 7, 8, 9, 26, 27). Alix conformation, interaction with binding partners, and activities are regulated by posttranslational modifications such as ubiquitination, phosphorylation, and palmitoylation (7, 19, 28, 29, 30, 31, 32). Cellular studies demonstrated that an interdomain contact between a Src-phosphorylation site in the Bro1 domain and the N terminus of the PRD keeps the protein in a closed, inactive conformation preventing its interaction with CHMP4 or retroviral Gag proteins (30, 33). Phosphorylation of Alix within the N-terminal portion of the PRD changes Alix from the closed to the open conformation, which in turn functions in cytokinetic abscission and retroviral budding (30). On the other hand, a biophysical study of monomeric Alix purified from insect cells suggested that the PRD does not affect the Bro1-mediated interaction between Alix and CHMP4 but rather inhibits the binding of Gag proteins (34). Recently, another biophysical study found no interaction between the N-terminal portion of the PRD and the Bro1 domain and instead showed that the hyperphosphorylated C terminus of the PRD binds to the anionic membrane-binding region of the Bro1 domain, causing autoinhibition of Alix interaction with the late endosomal membrane and its release into the cytosol (32). The apparent discrepancy between these studies could be explained by the use of purified Alix or its fragments in the biophysical investigation *versus* the use of cell extracts in the cellular studies that, as the authors propose, could involve Alix and/or CHMP oligomerization affecting their association (32, 34). Nevertheless, these reports highlight the importance of the PRD in the regulation of Alix interactions and functions. In this respect, it is noteworthy that the N-terminal portion of the PRD is the part of Alix that is recognized by the muscle-specific ubiquitin ligase complex CRL5^Ozz^ (previously known as Ozz-E3 ubiquitin ligase) (7, 35). Interaction of Alix with Ozz has also been shown to promote the open conformation of Alix by our group (7).

Alix exists as dimer or monomer (3, 13, 31), but it is still not clear which of these two forms engages in which of the protein’s numerous functions. Alix multimerization was shown to involve a short peptide (^852^PSYP^855^) at the C terminus of the PRD (36). A CHMP4-bound Alix dimer, mediated by the V domain, has been implicated in HIV-1 budding and MVB sorting of the activated EGFR (3, 13), while the ^852^PSYP^855^ motif has been found necessary for Alix’ role in virus infection (36), and HIV budding at the plasma membrane. Moreover, it has been proposed that, upon Bro1 domain–mediated binding to BMP at the membrane of late endosomes (37), Alix may dimerize to promote recruitment of ESCRT-III to regulate the formation of intraluminal vesicles in late endosomes/MVBs or exosomes (38).

A previous study has indicated that Alix functions at the interface between cytoskeleton and membranes (6). In line with this notion, we found that silencing Alix expression in C2C12 myoblast cell line affects the levels and distribution of F-actin, the formation of membrane protrusions, and the release of extracellular vesicles (7, 39). The connection between these two main roles of Alix became evident when we examined the consequences of Alix loss of function *in vivo*. The *Alix*^*−/−*^ mouse model develop, among other abnormalities, progressive and severe hydrocephalus (8). Mechanistically, we showed that loss of Alix affects the proper assembly and positioning of an actomyosin–tight junction complex at the apical side of adjacent epithelial cells that defines a spatial membrane domain essential for the maintenance of epithelial cell polarity and barrier (8). In this process, Alix plays the essential role of bridging membrane multiprotein complexes with the F-actin cytoskeleton (8).

Here, we have addressed the question of which form of Alix, dimeric *versus* monomeric, interacts with the F-actin cytoskeleton or components of membrane complexes that are routed to exosomes. We found that the levels of endogenous Alix dimer vary in different subcellular fractions, being enriched in exosomes and the cytoskeleton fraction. Alix dimerization occurs *via* disulfide bonds, engaging specifically Cys^814^ within the PRD that we identified as an essential residue for this process. We further show that by mutating Cys^814^ into Ser, an exclusively monomeric Alix, is proficient in loading syntenin into exosomes. On the other hand, ablation of Alix dimer impairs its interaction with F-actin, which in turn affects the remodeling of the actin cytoskeleton.

## Results

### Disulfide bond–linked alix homodimer is present in different subcellular compartments and in exosomes

**Figure 1:**
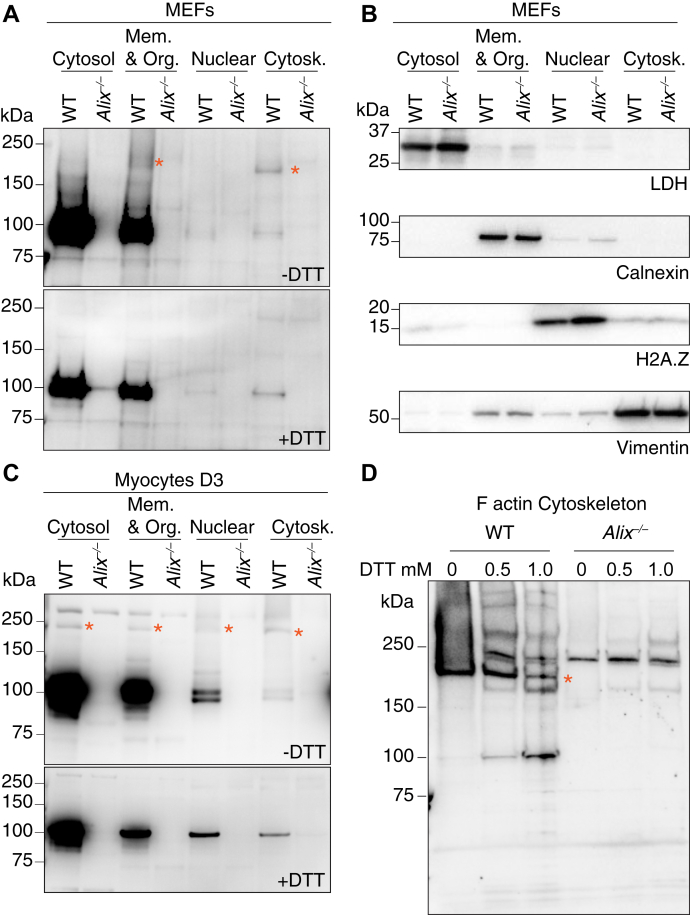
**Alix homodimers are linked *via* disulfide bond(s).***A*, endogenous Alix homodimer was revealed by Western blot in WT MEFs membrane & organelle (Mem. & Org.) and cytoskeleton (Cytosk.) fractions without reducing agent (asterisks; -DTT). 1 μg protein for cytosolic fraction and 6 μg proteins for others. *B*, subcellular fractionation verified by immunoblotting using antibodies against a cytosolic (Cytosol) fraction marker lactate dehydrogenase (LDH), an ER marker calnexin for membrane & organelle (Mem. & Org.) fraction, a nuclear fraction marker Histone H2A.Z (H2A.Z), and a cytoskeletal (Cytosk.) fraction marker vimentin. Three micrograms proteins were loaded for each fraction. *C*, Alix homodimer was shown by Western blot in WT differentiated myocytes (D3) in all four fractions without reducing agent (asterisks; -DTT). *D*, Alix Western blotting of the F-actin cytoskeleton fractions from WT, *Alix*^*−/−*^ MEFs in the presence of 0, 0.5, or 1.0 mM DTT. No more reducing agent was added before loading protein gels. Asterisk indicates the dimeric form of Alix. ER, endoplasmic reticulum; MEF, mouse embryonic fibroblast.

To test whether Alix dimerizes *via* disulfide bonds, subcellular fractions from WT and *Alix*^*−/−*^ mouse embryonic fibroblasts (MEFs) were separated on SDS polyacrylamide gels under reducing and nonreducing conditions, followed by immunoblotting with Alix antibody (Fig. 1*A* and Fig. S1*A*). Only in absence of the reducing agent, we could detect bands of ∼200 kDa, in addition to the Alix monomer of ∼100 kDa, in the membrane/organelle and, more evidently, in the cytoskeleton subcellular fractions. These high molecular weight forms were not visible in the *Alix*^*−/−*^ samples (Fig. 1*A* and S1*A*). In the presence of DTT, only the ∼100 kDa band was observed in the WT but not in the *Alix*^*−/−*^ fractions (Fig. 1*A* and S1*A*). The purity of the subcellular fractions was confirmed on immunoblots probed with antibodies against compartment-specific markers, that is, lactate dehydrogenase (LDH), calnexin, Histone H2A.Z, and vimentin (Fig. 1*B* and S1, *B*–*E*). Similar results were obtained using subcellular fractions from day 3 (D3) differentiated WT and *Alix*^*−/−*^ myocytes (Fig. 1*C* and S1, *F* and *G*). Under nonreducing conditions, a ∼200 kDa Alix band was evident in all four subcellular fractions of WT cells, together with the ∼100 kDa Alix monomer (Fig. 1*C* and S1*F*). Neither form of the protein was observed in the *Alix*^*−/−*^ myocytes, which confirmed their identity and the specificity of the Alix antibody. It is noteworthy that in myocytes, more so than in MEFs, monomeric Alix was resolved as a distinct doublet in absence of DTT. These two bands that may represent the close and open conformation of Alix were resolved as a single band under reducing conditions (Fig. 1*C* and S1*F*). These results indicate that Alix homodimer is formed by intermolecular disulfide bond(s) and Alix monomer may also keep its close conformation *via* an intramolecular disulfide link.

In both cell types, MEFs and myocytes, the majority of monomeric Alix was present in the cytosolic and membrane/organelle fractions. Alix dimer instead was distributed differently from its monomers, with the dimer/monomer ratio being highest in the cytoskeleton fraction (Fig. 1, *A* and *C* and Fig. S1, *A* and *F*), possibly reflecting distinct functions of the two forms of Alix in different subcellular compartments. In order to exclude the possibility that dimer formation was triggered by oxidation during the preparation of the samples, we isolated the Triton X-100 insoluble pellets from WT and *Alix*^*−/−*^ MEFs, representing the F-actin cytoskeleton fraction (40) in the presence of protective concentrations of the reducing agent DTT (0 mM, 0.5 mM or 1.0 mM) (41). Under these conditions, Alix dimers were still clearly visible (Fig. 1*D* and S1*H*), indicating that dimeric Alix is a physiological form of the protein.

**Figure 2:**
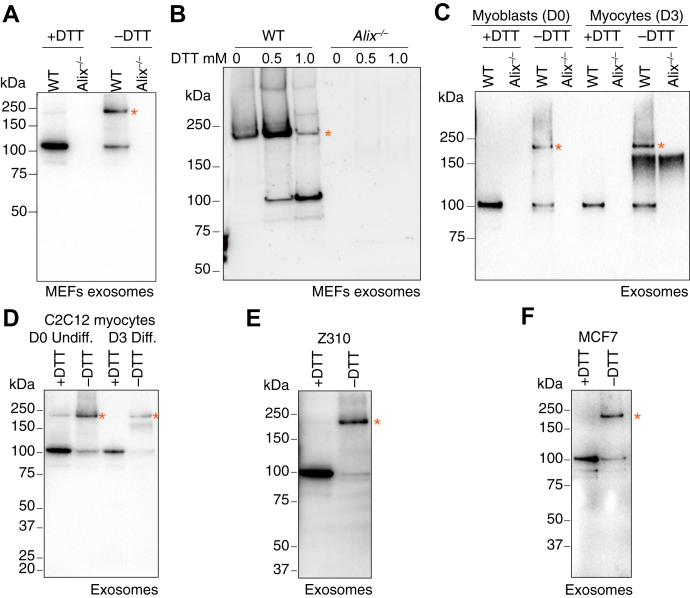
**Alix homodimers are enriched in exosomes from various cell types.***A*, Western blot analyses of crude exosomes isolated from WT and *Alix*^*−/−*^ MEFs probed with anti-Alix antibody. Asterisk marks the dimeric form revealed only under nonreducing condition (−DTT). *B*, exosomes isolated in the presence of 0, 0.5, or 1.0 mM final DTT concentration from WT and *Alix*^*−/−*^ MEFs cultures were subjected to Western blot analyses and probed with anti-Alix antibody. No more reducing agent was added before loading protein gels. Asterisk marks the dimeric form of Alix. *C*, WT and *Alix*^*−/−*^ proliferating myoblasts (D0) and differentiated (Diff.) myocytes (D3) were analyzed by Alix Western blotting under reducing (+DTT) and nonreducing (−DTT) conditions. Asterisks indicate the dimeric form revealed only under nonreducing condition. *D*–*F*, immunoblot analyses of crude exosomes isolated from mouse myoblast cell line C2C12 (*D*), rat choroid plexus epithelial cell line Z310 (*E*), and human breast cancer cell line MCF-7 (*F*) under reducing (+DTT) and nonreducing (−DTT) conditions and probed with anti-Alix antibody. Alix homodimers linked by disulfide bonds shown in nonreducing condition are indicated by asterisks and abolished by DTT. MEF, mouse embryonic fibroblast.

Alix dimerization was proposed to take place during the formation of intraluminal vesicles in late endosomes/MVBs or exosomes (38). We therefore examined whether endogenous Alix dimer was present in crude exosomes isolated from the culture medium of WT *versus Alix*^*−/−*^ MEFs. We found that this form of Alix was enriched in WT exosomes, separated on SDS gels under nonreducing conditions, but it was barely detected upon DTT treatment (Fig. 2*A* and Fig. S2*A*).

When WT exosomes were subjected to sucrose density gradient fractionation, both monomeric and dimeric forms of Alix sedimented in fractions 2 to 6 (corresponding to a density between 1.084–1.140 g/ml) (Fig. S2*B*), containing the exosome pool, as demonstrated by the sedimentation pattern of canonical exosome markers, CD9 and CD81 (Fig. S2, *C* and *D*). In addition, Alix dimer and monomer were also recovered in fractions 10 to 11 (Fig. S2*B*), which comprised the so-called membrane blebs and apoptotic bodies (42). Most importantly, when two concentrations of DTT (0.5 mM and 1.0 mM) were supplemented to the cell culture media upon collection for exosome isolation from WT and *Alix*^*−/−*^ MEFs, the Alix dimers remained clearly visible in exosomes (Fig. 2*B* and S2*E*). Dimeric Alix was also enriched in exosomes isolated from WT proliferating myoblasts and differentiated myocytes and sedimented primarily in fractions 2 to 6 (density between 1.084–1.140 g/ml) after sucrose density gradients (Fig. 2*C* and S3*A* and *B*). Both Alix monomer and dimer cofractionated with CD9 and CD81 after sucrose density gradients of exosomes from WT differentiated myocytes (D3) (Fig. S3, *C* and *D*). We also confirmed the presence of dimeric Alix in the mouse myoblast cell line C2C12, the rat choroid plexus epithelial cell line Z310 (43) and the human breast cancer cell line MCF-7 (Fig. 2, *D*–*F* and Fig. S3, *E*–*G*), indicating its broad distribution across different mammalian cell types.

### Cys^814^ in the PRD mediates alix dimerization

**Figure 3:**
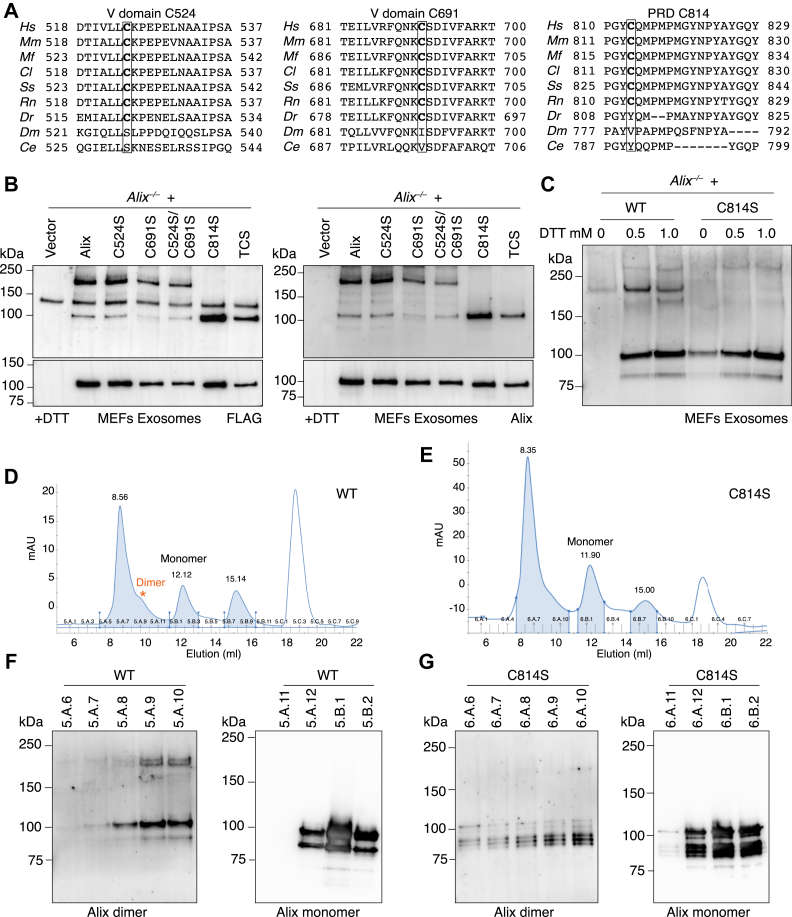
**Cys**^**814**^**in the PRD mediates Alix dimerization.***A*, conservation of the Cys^524^, Cys^691^, and Cys^814^ (bold and boxed) among mammals. *Hs*: human; *Mm*: mouse; *Mf*: Macaque monkey; *Cl*: dog; *Ss*: pig; *Rn*: Rat; *Dr*: Zebrafish; *Dm*: fly; *Ce*: worm. The Cys^524^, Cys^691^, and Cys^814^ are shown in bold and box. *B*, Western blotting of the exosomes purified from *Alix*^*−/−*^ MEFs transduced by empty vector or MSCV expressing N-terminal FLAG-tagged full-length Alix or cysteine mutants harboring C524S, C691S, C524S/C691S, C814S, C524S/C691S/C814S (TCS) under nonreducing (−DTT) and reducing conditions (+DTT) probed with antibodies against FLAG or Alix. *C*, exosomes isolated from *Alix*^*−/−*^ MEFs transduced by MSCV expressing N-terminal FLAG-tagged full-length Alix WT or C814S in the presence of 0, 0.5, or 1.0 mM DTT were subjected to Western blot analyses and probed with anti-Alix antibody. *D*, chromatography profile of the recombinant WT Alix proteins purified from human Expi293F cells in the presence of 1 mM DTT. *Red* asterisk marks the distinct shoulder. *E*, chromatography profile of the recombinant Alix (C814S) proteins purified from human Expi293F cells in the presence of 1 mM DTT. *F*, Alix Western blots of SEC fractions to show that dimeric WT Alix was eluted in fractions 5.A.9 and 5.A.10 consistent with the SEC profile. *G*, Alix Western blots of SEC fractions to show that dimeric form was missed for Alix (C814S) proteins in corresponding high molecular weight fractions. MEF, mouse embryonic fibroblast; MSCV, Murine stem cell virus; PRD, proline-rich domain; SEC, size-exclusion chromatography.

We next investigated the mode of Alix dimerization mediated by disulfide bonds, focusing on cysteine residues located in the V domain and the PRD of the full-length protein. The V domain has two cysteines at amino acids 524 and 691, while the PRD has only one cysteine at amino acid 814. All three residues are conserved in mammalian homologs of Alix, such as human, monkey, dog, pig, mouse, and rat, identified in a NCBI BLAST search (Fig. 3*A*).

To map out which of these residues is responsible for disulfide bond formation and dimerization, all three cysteines in the full-length Alix were mutated, individually or in combination, to serine. Each mutant was expressed in *Alix*^*−/−*^ MEFs, and exosomes were isolated from the cultured media. Under nonreducing conditions, the Alix variants carrying the individual mutations C524S and C691S or the double mutations C524S/C691S formed dimers (Fig. 3*B* and S4, *A*–*C*). In contrast, the Alix variants harboring the C814S mutation alone or the triple mutations C524S/C691S/C814S (TCS) could no longer form dimers (Fig. 3*B* and S4, *A*–*C*). Notably, upon supplementation of 0.5 or 1 mM DTT to the culture medium prior to exosome isolation, WT Alix still formed dimers, while the C814S mutant protein did not under any condition (Fig. 3*C* and S4*D*). Thus, Cys^814^ within the PRD is essential for the formation of disulfide bond–linked Alix homodimer.

To unequivocally prove the physiological existence of Cys^814^-mediated Alix dimers, we have overexpressed an N-terminally FLAG-tagged WT Alix and Alix (C814S) recombinant proteins in human EXpi293F cells. The overexpressed proteins were purified using anti-FLAG affinity gel, and the different forms of Alix were separated by size-exclusion chromatography (SEC) in the presence of 1 mM DTT. The chromatography profile showed that most of the WT or Alix (C814S) mutant were eluted from fractions 5.A.12 to 5.B.2 or fractions 6.A.11 to 6.B.2, respectively, corresponding to the monomeric form (Fig. 3, *D* and *E* and Fig. S4*E*). However, the SEC profile of WT Alix showed a potentially distinct shoulder around fraction 5.A.9 to 5.A.10, which was absent in the Alix (C814S) elution profile and could represent dimeric Alix (see asterisk in Fig. 3*D*). This assumption was confirmed by Western blot analyses of the individual fractions that detected a prominent dimeric form of WT Alix in exactly the same two fractions, which was absent in the corresponding fractions of the C814S mutant. (Fig. 3, *F* and *G* and Fig. S4, *F* and *G*).

### Monomeric Alix is efficient in loading syntenin into exosomes

**Figure 4:**
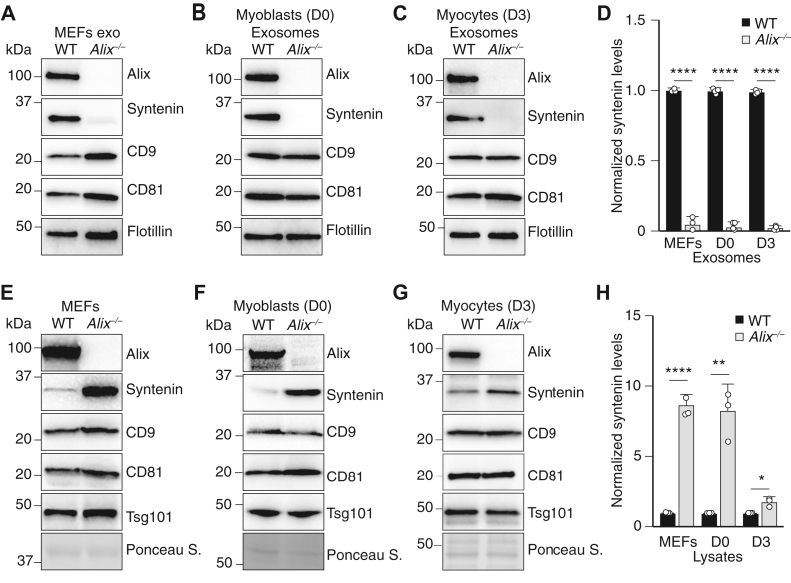
**Alix is essential for loading syntenin into exosome and modulates its homeostasis.***A*–*C*, crude exosomes (exo) isolated from WT and *Alix*^*−/−*^ MEFs (*A*), proliferating D0 myoblasts (*B*) and differentiated (Diff.) D3 myocytes (*C*) were examined on immunoblots probed with antibodies against established exosomal markers Alix, syntenin, CD9, CD81, and Flotillin, the last one used as the loading control (14). *D*, quantification of syntenin from (*A*–*C*). *E*–*G*, corresponding total cell lysates from MEFs (*E*), proliferating D0 myoblasts (*F*), and differentiated (Diff.) D3 myocytes (*G*) were analyzed on immunoblots probed with antibodies against established exosomal markers Alix, syntenin, CD9, CD81, and Tsg101, Ponceau S was used as the loading control. *H*, quantification of syntenin from (*E*–*G*). Values are expressed as means ± SD. Statistical analyses was performed using the student *t* test; ∗ *p* < 0.05, ∗∗ *p* < 0.01, ∗∗∗∗*p* < 0.0001. MEF, mouse embryonic fibroblast.

In order to define a role of dimeric Alix in exosome biogenesis and cargo loading, we first ascertained the proteomic profiles of exosomes isolated from the culture medium of *Alix*^*−/−*^ MEFs, as well as proliferating myoblasts (day 0), and differentiated myocytes (day 3) derived from *Alix*^*−/−*^ skeletal muscle. The results of these analyses revealed that syntenin was the only exosomal marker that was dramatically reduced or absent in *Alix*^*−/−*^ exosomes compared to WT exosomes (Table S1) regardless of their cell derivation. The levels of other canonical exosome markers, such as CD9, CD81, and Tsg101 (Table S1), remained largely unchanged in absence of Alix. Immunoblot analyses of these exosomal preparations validated the proteomic data, showing more than 95% reduction in the levels of syntenin in *Alix*^*−/−*^ exosomes from all three cell cultures compared to the levels in WT exosomes (Fig. 4, *A*–*D*, Fig. S5, *A*–*O* and Fig. S6, *A*–*C*). In WT MEF’s exosomes, syntenin cofractionated with Alix in fractions corresponding to densities of 1.084 to 1.140 g ml^-1^ (44) after sucrose density gradient centrifugation, but it was not detected in the corresponding fractions of *Alix*^*−/−*^ exosomes, although a small portion of the protein sedimented in fractions containing membrane blebs or apoptotic bodies (42) (Fig. S6*D*). Similar results were obtained after sucrose density gradient centrifugation of exosomes from differentiated myocytes (Fig. S6*E*). The lack of syntenin in *Alix*^*−/−*^ exosomes was accompanied by accumulation of the protein in total cell lysates of *Alix*^*−/−*^ MEFs compared to WT MEFs, while other exosome markers showed no significant difference in levels (Fig. 4*E*, S6, *F*–*L*). We also ascertained that the mRNA levels of syntenin were not altered in *Alix*^*−/−*^ cells, as determined by RT-PCR (Fig. S7*A*), suggesting that its intracellular accumulation was caused by failed trafficking of a pool of the protein to exosomes and not by altered transcription. A similar distribution pattern of syntenin was seen in *Alix*^*−/−*^ proliferating myoblasts and differentiated myocytes (Fig. 4, *F*–*H*; Fig. S7, *B*–*O*), implying a conserved mechanism. Together, these data reinforce the notion that deficiency of Alix *in vivo* results in impaired loading of syntenin into exosomes, making this parameter an ideal readout to assess the function of dimeric/monomeric Alix in exosomes of deficient cells.

**Figure 5:**
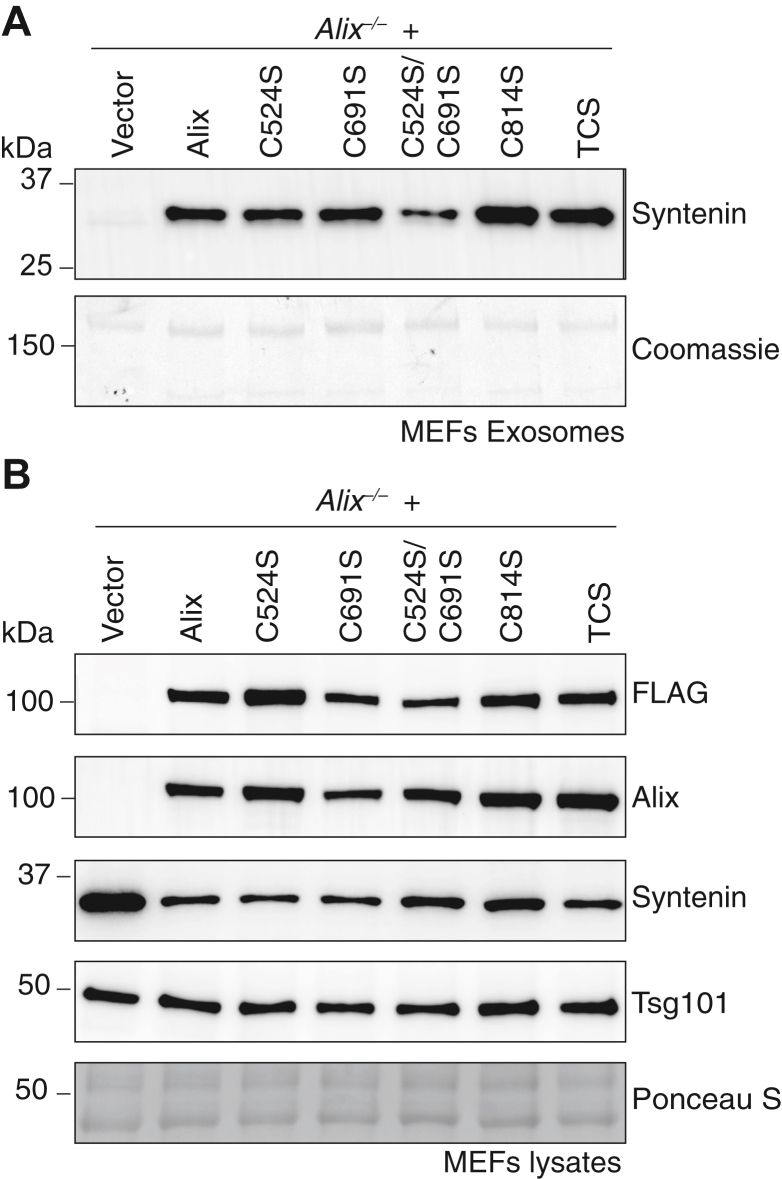
**Monomeric Alix is efficient in loading syntenin into exosomes.***A*, Western blotting of the exosomes isolated from *Alix*^*−/−*^ MEFs transduced by empty vector or MSCV expressing N-terminal FLAG-tagged full-length Alix or cysteine mutants harboring C524S, C691S, C524S/C691S, C814S, C524S/C691S/C814S (TCS) probed with antibodies against syntenin. Coomassie staining was used as the loading control. *B*, corresponding total cell lysates were analyzed by Western blotting for specific proteins using cognate antibodies. Ponceau S was used as the loading control. MEF, mouse embryonic fibroblast; MSCV, Murine stem cell virus.

We therefore examined the recruitment of syntenin into exosomes derived from *Alix*^*−/−*^ MEFs expressing either WT Alix or Alix variants harboring single or combined mutations of the cysteine residues: Cys^814^ or Cys^524^ and Cys^691^. The Alix C524S or C691S mutant, as well as the double mutant C524S/C691S, were still proficient in loading at least a portion of syntenin into exosomes (Fig. 5*A* and Fig. S8*A*). However, the level of the protein was clearly reduced in exosomes derived from the C524S/C691S culture (Fig. 5*A* and S8*A*), likely due to the position of the two cysteine residues within the second arm of the V domain, which may influence the binding of syntenin, centered on Phe^676^ (14). Notably, the Alix C814S and the TCS variants, which could not form homodimers, were fully capable to deliver syntenin into exosomes (Fig. 5*A* and S8*A*), suggesting that monomeric Alix is the form of the protein that loads syntenin into exosomes. Consistent with these results, the levels of the syntenin protein in total lysates of cells expressing the Alix C814S variant was reduced compared to its levels in *Alix*^*−/−*^ MEFs mock transduced with an empty vector (Fig. 5*B* and S8, *B*–*F*). These results indicate that abrogating Alix dimerization does not compromise the loading of a syntenin pool into exosomes.

### The Bro1 domain directs alix into exosome while the V domain is required for the loading of syntenin

**Figure 6:**
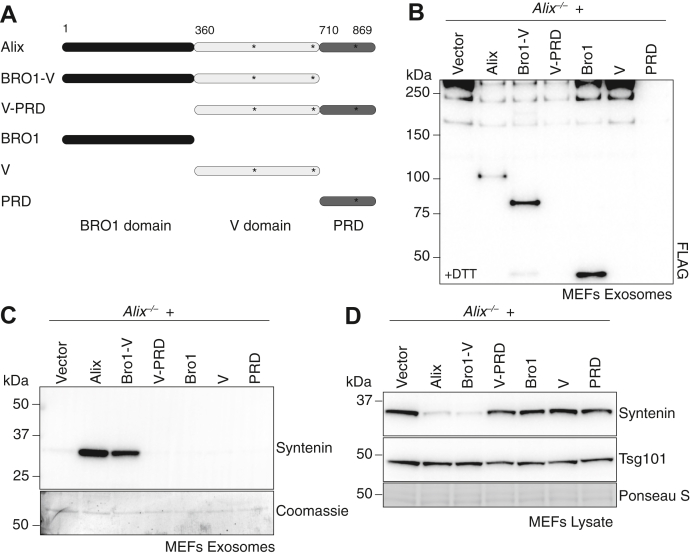
**Bro1 directs Alix to be loaded to exosome and V is required for loading syntenin into exosomes.***A*, schematic representation of full-length Alix or its truncated fragments. Numbers indicate residue positions. Asterisks indicate the position of the three cysteines. *B*, Western blot analysis using anti-FLAG antibody of the exosomes isolated from *Alix*^*−/−*^ MEFs, which were transduced with empty vector control or MSCV expressing N-terminal FLAG-tagged full-length Alix or its truncated fragments. *C*, syntenin Western blot analysis using the exosomes isolated from *Alix*^*−/−*^ MEFs, which were transduced with empty vector control or MSCV expressing N-terminal FLAG-tagged full-length Alix or its truncated fragments (Bro1-V, V-PRD, Bro1, V, or PRD). Coomassie staining was used as the loading control. *D*, corresponding total cell lysates were analyzed by Western blotting using antisyntenin and anti-Tsg101 antibodies. Ponceau S was used as the loading control. MEF, mouse embryonic fibroblast; MSCV, Murine stem cell virus.

We next evaluated the role of the three domains of Alix in sorting the protein into exosomes and in loading syntenin into exosomes by using *Alix*^*−/−*^ MEFs transduced with N-terminal FLAG-tagged full-length Alix or its various fragments, Bro1, Bro1-V, V, V-PRD, and PRD (Fig. 6, *A* and *B* and Fig. S9*A*). *Alix*^*−/−*^ MEFs expressing full-length Alix restored the traffic of syntenin into exosomes, which was accompanied by reduction of the levels of this protein in the corresponding cell lysates (Fig. 6, *C* and *D* and Fig. S9, *B*–*F*). Among the truncated fragments, only the Bro1 and Bro1-V retained the capacity to traffic to the exosomes (Fig. 6*B* and S9*A*), demonstrating that Bro1 is essential and sufficient for Alix sorting into these vesicles (11, 32, 37, 45). However, only Bro1-V could load syntenin into exosomes and reduced the intracellular levels of the protein, confirming the requirement of the V domain for this process (14) (Fig. 6, *C* and *D* and Fig. S9, *B*–*F*). It is noteworthy that full-length Alix was more efficient in loading syntenin into exosomes than the truncated Bro1-V, despite the latter was expressed in seemingly higher amounts (Fig. 6, *B* and *C* and Fig. S9, *A*–*C*), indicating that Bro1-V conformation differs from that of the full-length protein.

### Alix dimer interacts with F-actin and promotes cytoskeleton reassembling

**Figure 7:**
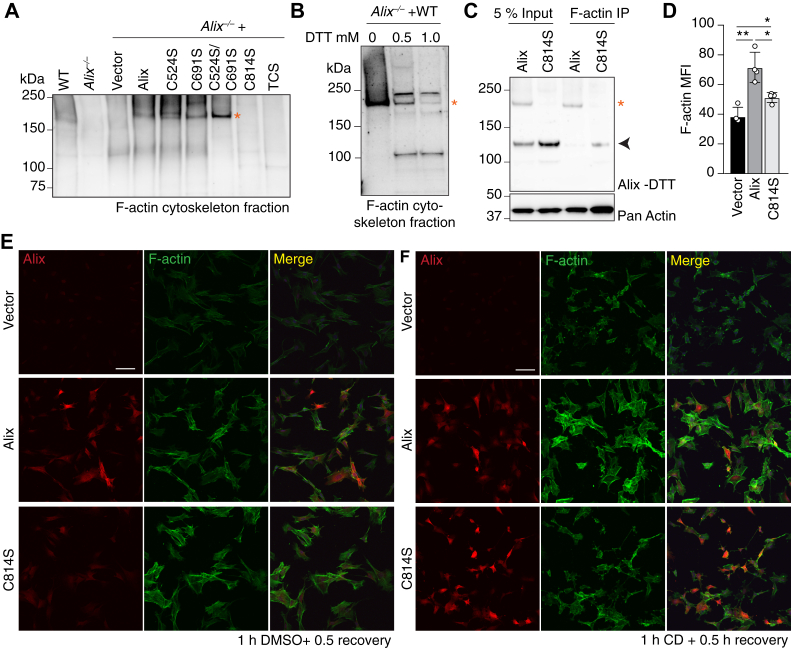
**Alix dimers interact with F-actin cytoskeleton and modulate its reassembly**. *A*, Alix Western blotting of the F-actin cytoskeleton fractions from WT, *Alix*^*−/−*^ MEFs, and *Alix*^*−/−*^ MEFs transduced by empty vector or MSCV expressing N-terminal FLAG-tagged full-length Alix or mutants harboring C524S, C691S, C524S/C691S, C814S, C524S/C691S/C814S (TCS) under nonreducing (−DTT) condition to examine dimers. Asterisk indicates the dimeric form of Alix. *B*, Alix Western blotting analysis of F-actin cytoskeleton fractions from *Alix*^*−/−*^ MEFs transduced by MSCV expressing N-terminal FLAG-tagged Alix WT in the presence of 0, 0.5, or 1 mM DTT. No more reducing agent was added before loading protein gels. Asterisk marks the dimeric form of Alix. *C*, Alix dimer coprecipitates with F-actin. IP, immunoprecipitate. Asterisk indicates dimeric form. Arrowhead marks unspecific protein bands. *D*, quantification of the mean fluorescence intensity (MFI) of F-actin (*n* = 4 fields) after treatment with cytochalasin D (CD) for 1 h and allowed to recover for 0.5 h. Values are expressed as mean ± SD. Statistical analyses was performed using the Brown–Forsythe test; ∗ *p* < 0.05, ∗∗ *p* < 0.01. *E* and *F*, *Alix*^*−/−*^ MEFs transduced with MSCV expressing WT Alix or Alix harboring C814S mutant (C814S). Cells were treated with DSMO for 1 h (control) (*E*) or 10 μM cytochalasin D (CD) (*F*) for 1 h and allowed to recover for 0.5 h before immunofluorescent staining with anti-Alix antibody (*red*) and Alexa Fluor 488 dye conjugated phalloidin to probe F-actin (*green*). The scale bars represent 100 μm. MEF, mouse embryonic fibroblast; MSCV, Murine stem cell virus.

Given that the Alix dimer is enriched in cytoskeleton fractions and that Alix physically interacts with actin (9), we wanted to investigate whether abrogating dimer formation would affect this binding. To test this idea, we first isolated the Triton X-100 insoluble pellets, representing the F-actin cytoskeleton fraction, from *Alix*^*−/−*^ MEFs transduced with Murine stem cell virus (MSCV)–based vectors, expressing either WT Alix or Alix variants harboring the aforementioned cysteine mutations. On Western blots, Alix dimers were preferentially detected in the F-actin cytoskeleton fractions from *Alix*^*−/−*^ MEFs expressing either WT Alix or Alix (C524S), Alix (C691S), Alix (C524S/C691S) (Fig. 7*A* and Fig. S10*A*). Again, no dimeric Alix was present in cells expressing either Alix (C814S) or TCS, confirming that dimer formation depends on Cys^814^ (Fig. 7*A* and S10*A*). Even when the F-actin cytoskeleton fraction was isolated in the presence of 0.5 mM or 1.0 mM DTT, Alix Cys^814^-mediated dimers were still clearly visible (Fig. 7*B* and S10*B*). Together, these data suggest that F-actin interacts preferentially with the Alix dimer. To prove this hypothesis, the F-actin cytoskeleton fractions from *Alix*^*−/−*^ MEFs expressing either WT Alix or the Alix (C814S) variant were immunoprecipitated with F-actin antibody and probed on immunoblots with Alix and pan actin antibodies. The results unequivocally showed that F-actin coimmunoprecipitated preferentially with the Alix dimer (Fig. 7*C* and S10*C*).

We previously demonstrated that the reassembly of F-actin filaments after depolymerization with cytochalasin D was defective in Z310 choroid plexus epithelial cells silenced for Alix (8). We now tested whether Alix dimer plays a role in the F-actin reassembly process. *Alix*^*−/−*^ MEFs expressing either WT Alix or the Alix (C814S) variant were compared with empty vector transduced *Alix*^*−/−*^ MEFs. All cultures were treated with cytochalasin D for 1 h, a period that provoked extensive disassembly of the F-actin cytoskeleton that resulted in rounding up of the cells and loss of normal morphology in all cultures stained with phalloidin (Fig. S10*D*). Cells were then allowed to recover for 30 min after removal of the drug. Based on the phalloidin staining of the recovered cultures, an overall reduction of the mean cellular F-actin content was measured in *Alix*^*−/−*^ MEFs transduced with the empty vector or with the vector expressing the Alix (C814S) variant, when compared with cells expressing the WT protein (Fig. 7, *D*–*F*). This result underlines the need for dimeric Alix to restore F-actin levels and to reassemble the actin cytoskeleton. In addition, a large percentage of *Alix*^*−/−*^ MEFs transduced with the empty vector (n = 186) or with the Alix (C814S) variant (n = 284) showed abnormal cell morphology after the 30 min recovery time (Fig. 7*F*). In contrast, the majority of *Alix*^*−/−*^ MEFs expressing WT Alix (n = 331) recovered after treatment, displaying normalized F-actin content and assembly, as well as normal cell morphology (Fig. 7, *D*–*F*). Taken together, our data demonstrate that dimeric Alix is the form that associates with the F-actin cytoskeleton and promotes its remodeling.

## Discussion

Our current study has uncovered distinct functional roles of Alix dimer *in vivo*. This form of the protein is ubiquitously present together with Alix monomer in different cell types and across mammalian species. However, the ratio between dimeric and monomeric Alix varies among cell types and subcellular localizations, being most abundant in the cytoskeleton fractions and in exosomes, which suggests specific functions for Alix dimer in these two compartments.

We reveal a novel mode of dimer formation that is mediated by disulfide bonds and requires Cys^814^, the only cysteine residue localized in the Alix PRD. PRDs are one of the most frequently occurring signaling modules in eukaryotic proteins (4). Their disordered structure favors dynamic interactions with a variety of proteins and protein complexes. In addition, PRDs present in intrinsically disordered proteins contribute to protein–protein interaction during the process of solution-to-gel phase separation (46). Alix PRD retains similar characteristics (5), being responsible for binding to multiple protein partners, such as TSG101, Src SH3 domain, ALG-2, Endophilin, SETA/CIN85, actin, CRL5^Ozz^, and others (7, 9, 15, 16, 17, 18, 19). It is also susceptible to posttranslational modifications, like phosphorylation (30). However, the structural features of Alix PRD made it difficult to study it as a separate fragment or in the context of the full-length protein. In a recent study, investigators have succeeded to produce the Alix PRD *in vitro* by dividing it into two separate fragments (5). This approach enabled the biophysical characterization of this domain. These authors found that the disordered N-terminal portion of Alix PRD is soluble and contains three Pro-rich motifs that create multiple interaction sites for TSG101; instead, the Tyr-rich C-terminal portion where Cys^814^ resides, which was reported earlier to be involved in Alix multimerization, was shown to form reversible amyloid (5, 36). In line with this finding, we detected bands/smears of >200 kDa, positive for Alix in WT MEFs and D3 myocytes under nonreducing conditions (Fig. 1, *A* and *C*), as well as in their corresponding exosomes (Fig. 2, *A* and *C*). Neither *Alix*^*−/−*^ samples nor WT samples treated with DTT displayed these bands/smears, indicating that they are likely caused by Alix multimerization mediated by disulfide bonds. These multimers are always observed together with Alix homodimer, suggesting that Alix dimerization is required for its multimerization. Our finding that Cys^814^ within the PRD is essential for dimer formation, underlines a regulatory role for this domain in Alix dimerization/multimerization to coordinate specific functions of Alix, namely F-actin reassembling and exosomal cargo loading.

It was reported earlier that during budding HIV virus hijacks, the Alix-mediated mechanism of membrane remodeling, which is normally used to recruit cellular proteins, like syntenin, into exosomes. During virus budding, the *Gag* gene encodes two C-terminal late domains with the highly conserved sequences PTAP and LYPXnL that recruit Tsg101 and Alix, respectively (22, 47). These two domains and their cognate cellular partners operate in a redundant way to support virus release, independently (48, 49). In contrast, we found that ablation of Alix *in vivo* does not influence the levels of Tsg101, rather syntenin is no longer delivered to exosomes, implying that Alix is the only protein mandatory for this process. Furthermore, we demonstrate that the monomeric form of Alix is sufficient to load syntenin into exosomes. Thus, the presence of Alix dimer in exosomes might have an independent function from cargo loading and instead may be needed for exosome biogenesis, as hypothesized previously (38).

Syntenin is a multifunctional adapter protein that interacts with a variety of protein partners involved in many cellular processes, including synapse formation, protein trafficking, neuronal morphogenesis, and exosome biogenesis (50, 51). Our data imply that loading of syntenin into exosomes may serve as a regulatory mechanism to control the total pool of cellular syntenin. This might be particularly relevant in the context of neurodegenerative diseases, including Alzheimer’s disease, and cancer (50, 51). Syntenin expression is upregulated in several cancers, and preclinical studies have shown that inhibiting syntenin either genetically or pharmacologically suppresses cancer metastasis (52). It has been hypothesized that exosomal syntenin contributes to metastatic spread (53) because tumor cell (donor)–derived exosomes induce migration of endothelial HUVEC cells (recipient), while exosomes deprived of syntenin are significantly less promigratory (53). Thus, it is tempting to speculate that inhibiting Alix–syntenin interaction pharmacologically could be a plausible approach to block syntenin loading into exosomes so that to interfere with the metastatic process.

The ability of Alix protein to interact with actin and in turn modulate actin cytoskeleton homeostasis has been reported by a few groups, including ours (6, 7, 8, 9, 27). We now provide evidence that it is exclusively the dimeric form of Alix that interacts with F-actin and regulates its reassembly, assigning a specific role of Alix dimer within the actin cytoskeleton. This notion is particularly relevant in processes that involve both membrane remodeling and F-actin cytoskeleton. In this respect, a recent study showed that CCL2, a chemokine induced upon HIV-1 infection, mobilizes Alix from F-actin to facilitate Gag-p6 mediated HIV-1 virion release (26). It appears that HIV-1 can seize Alix from F-actin compartment through CCL2 to function in membrane remodeling for virion release. A wide range of enveloped viruses, including HIV-1 and other retroviruses, as well as additional RNA and DNA viruses embed the LYPXnL motif in their late assembly domain that is recognized by Alix (54). Moreover, viruses are known to modify and modulate the host cell actin cytoskeleton, affecting every stage of the viral life cycle from entry to assembly and egress (55). It would be interesting to investigate whether Alix dimers play a role in any of these processes.

In summary, this study reveals that endogenous Alix forms dimers by disulfide bonds involving the PRD. This dimerization adds a tier to the regulation of Alix interactions with one of its protein partners, F-actin, and therefore influences downstream cellular processes. Further investigations might elucidate whether dimeric Alix modulates other functions of this multivalent protein.

## Experimental procedures

### Mice

Mice of all genotypes were accommodated in the pathogen-free Animal Resource Center at St Jude Children’s Research Hospital. WT mice were C57Bl/6xDBA2 (B6D2F1; Jackson Labs); *Alix*^*−/−*^ mice carry the homozygous deletion of *Alix* on a C57Bl/6 background (8). Animals were housed in a fully Assessment and Accreditation of Laboratory Animal Care (AAALAC) accredited animal facility. All mouse procedures were performed in compliance with our animal protocols approved by the St Jude Institutional Animal Care and Use Committee and NIH guidelines.

### Antibodies

We used the following commercial antibodies for Western blot analyses: anticalnexin (Sc-6465), anti-CD81 (Sc-166029), and anti-Tsg101 (Sc-7964) from Santa Cruz Biotechnology; anti-CD9 (553758) and anti-Flotillin (610820) from BD Biosciences; anti-FLAG (F3165) from Sigma; anti-Histone H2A.Z (2718) from Cell Signaling; antilactate dehydrogenase (AB1222) from Millipore; and antivimentin (20R-VP004) from Fitzgerald. The antisyntenin antibody used in this study was affinity purified further from antisyntenin (Santa Cruz Biotechnology, sc-48742). We used anti-F-actin (abcam, ab205) for coimmunoprecipitation.

### Cell transduction

Alix, its fragments, or cysteine mutants were cloned into the MSCV-based bicistronic retroviral vector MSCV-IRES-YFP (56). All constructs were sequenced to ensure successful desired clonings. Ecotropic retroviruses were generated by transfecting the packaging cell line Phoenix Eco with constructs using the transfection reagent FuGENE 6 purchased from Promega. *Alix*^*−/−*^ MEFs were then transduced with retroviruses. Two or three days after transduction, YFP+ cells were sorted by fluorescence-activated cell sorting. Sorted cells were maintained in culture.

### Cell culture

MEFs, muscle neonatal fibroblast cells, cell lines Z310 and MCF-7 were maintained in Dulbecco’s modified Eagle’s medium (DMEM) and supplemented with 10% fetal bovine serum (Life Technologies), 2 mM Glutamax, penicillin (100 U/ml), and streptomycin (100 mg/ml). Mouse myoblast cell line C2C12 was cultured in DMEM and supplemented with 15% fetal bovine serum (Life Technologies), 2 mM Glutamax, penicillin (100 U/ml), and streptomycin (100 mg/ml). Mouse gastrocnemius (GA) and soleus muscle were collected (57) to be used to isolate myoblasts. Myoblast cultures were established as previously described (58, 59, 60) and maintained in F10 medium supplemented with basic fibroblast growth factor (b-FGF), L-glutamine, and 20% fetal bovine serum (Life Technologies) on collagen-treated dishes (BD Biosciences). Differentiation was induced when the medium was changed to DMEM supplemented with L-glutamine and 2% horse serum in place of fetal calf serum (differentiation medium). All cells were confirmed to be negative for myoplasma.

### Subcellular fractionation

Fresh cell pellets were fractionated using a ProteoExtract Subcellular Proreome Extraction Kit (Calbiochem, VWR International Ltd) following the manufacturer's user protocol. The purity of each fraction was checked by immunoblotting using antibodies against cytosolic fraction marker lactate dehydrogenase (LDH), endoplasmic reticulum marker calnexin, nucleic fraction marker Histone H2A.Z, and cytoskeletal fraction marker vimentin.

### Western blot analysis

For Western blot analyses under reducing condition (+DTT), the final concentration of 16.7 mM DTT was included in 1 × Laemmli sample buffer with protein samples and heated for 4 min at 95 °C before electrophoresis. For Western blot analyses under nonreducing conditions (-DTT), no reducing agent was added. The protein samples were mixed with Laemmli sample buffer and heated for 4 min at 95 °C before electrophoresis. Proteins were transferred onto polyvinylidene difluoride membrane (Millipore). Membranes were blocked with 3% bovine serum albumin in Tris-buffered saline (TBS) with 0.05% Tween 20 for at least 1 h at room temperature (RT) and incubated with the appropriate antibodies in 3% bovine serum albumin in TBS with 0.05% Tween 20 overnight at 4 °C. All primary antibody incubations were followed by incubation with secondary horseradish peroxidase–conjugated antibody (Jackson ImmunoResearch) in 3% bovine serum albumin in TBS with 0.05% Tween 20 and visualized using Femto chemiluminescent substrate (Thermo Scientific) and ChemiDoc MP Imaging System (Bio-Rad).

### Crude exosomes isolation and proteomics

Cells were washed with PBS twice and then cultured for another day in medium containing the serum, which had been centrifuged at 100,000×*g* for overnight at 4 °C to deplete contaminating vesicles. Tissue culture supernatants were collected and centrifuged for 10 min at 300 × *g*, 10 min at 1200 × *g*, and for 30 min at 10,000 × *g* to remove cellular debris. Vesicles were spun down by ultracentrifugation at 100,000 × *g* for 2 h at 4 °C. Supernatants were discarded and vesicles were washed in PBS. Crude exosomes were collected by centrifugation at 100,000 × *g* for 2 h at 4 °C and then resuspended in PBS. Quantification of the resulting crude exosomes was done using the bicinchoninic acid method.

For proteomics study, 6 μg fibroblast exosomes, 10 μg proliferating myoblast exosomes, or D3 myocyte exosomes were subjected to mass spectrometry and analyzed with MaxQuant at Proteomics Center, Erasmus Medical Center, The Netherlands. For crude exosomes isolated from MEFs cultures in the presence of DTT, 0, 0.5, or 1.0 mM DTT in final concentration were added to tissue culture supernatants harvested 24 h after starting the exosome collection. About 0, 0.5, or 1.0 mM DTT in final concentration were also included in PBS for washing and resuspending exosomes. No extra reducing agent was added before loading protein gels for Western blot analyses.

### Sucrose density gradient fractionation

Crude exosomes were resuspended in 10 mM pH 7.4 TBS containing 0.25 M sucrose and loaded on to the top of a step gradient comprising layers of TBS containing 0.25 to 2 M sucrose,1 mM Mg(Ac)_2_, and a cocktail of protease inhibitors (Roche) (42, 60, 61, 62, 63). The gradients were centrifuged at 100,000 × *g* for 2.5 h using a Beckman SW41 Ti rotor. Eleven 1 ml fractions were collected starting from the top of the gradient to bottom and precipitated with 100 % trichloroacetic acid (final concentration 10%). Samples were analyzed by SDS/PAGE and Western blotting as described.

### Recombinant protein purification and SEC

For recombinant protein expression of Alix WT and Alix C814S, human Expi293F cells (2.75 × 10^6^ cells/ml) were transiently transfected with 1 mg of Alix WT and Alix C814S mutant DNA, and the transfected cells were incubated at 37 °C for 60 h in an orbital shaker at 140 rpm with 5% CO2. Cells were harvested by centrifugation (1000*g* for 15 min), resuspended in buffer A (Tris 50 mM pH 7.6, 200 mM NaCl, 1 mM DTT) supplemented with one complete protease inhibitor cocktail EDTA-free tablet (SKU 5056489001) from Roche, and sonicated with five pulses for 30 s with EPSON 4C15 robotics sonicator. Cell lysate was centrifuged at 20,000 rpm for 30 min at 4 °C. The cleared lysate was applied to pre-equilibrated anti-FLAG M2 affinity gel (A2220) from Sigma and incubated for 1 h at 4 °C. The incubated resin was collected with centrifugation at 1500 rpm for 5 min at 4 °C and washed with buffer A. After extensive washing, the FLAG-tagged protein was eluted using 3 × FLAG peptide (St Jude Peptide synthesis facility) and elution fractions were subjected to SDS-PAGE. After confirming the band on SDS-PAGE, protein was injected onto pre-equilibrated Superdex 200 increase 10/300 Gl column (GE Healthcare) at a flow rate of 0.5 ml/min, and gel filtration fractions were collected in a 96-well deep well block (Thermo Scientific) for Western blot analysis.

### Quantitative real time PCR

Total RNA was isolated from WT and *Alix*^*−/−*^ MEFs by using the PureLink RNA kit (LifeTechnologies) according to manufacturer’s protocol. DNA contaminants were removed by on column DNAse I treatment (Life Technologies), according to the manufacturer’s protocol. RNA quantity and purity were measured using Nanodrop Lite spectrophotometer (Thermo Scientific). Complementary DNA (cDNA) was produced using 3 μg total RNA with iScript Advanced cDNA synthesis kit (Bio-Rad). Quantitative real time PCR was performed using SsoAdvanced Universal SYBR green supermix (Bio-Rad) (12.5 μl), 1 μl (50 ng) of cDNA, 10 μM primer (Alix FW: TAG TGT TTG CAC GGA AGA CAG, Alix RV: GGG AGG ACT GAT AGG CTG GA, syntenin FW: TCA CCA TGA CGA TCC GTG AC, syntenin RV: CCGTTG ATC TCA CAG ATG TGG), and RNAse-free water in a 25 μl reaction volume on a CFX96 real time PCR Machine (BioRad). Samples were normalized to murine 18S rRNA (catalog no.: #PPM57735E, SABiosciences).

### F-actin cytoskeleton fraction isolation

MEFs were cultured following cell culture protocol mentioned previously. After being trypsinized and washed once with PBS, MEFs pellets were resuspended in ice-cold hypotonic buffer (10 mM Tris–HCl, pH 7.8, 1 mM CaCl_2_, 5 mM KCl, and a cocktail of phosphatase and protease inhibitors without EDTA (Roche)), and incubated for 15 min on ice (59). Nuclear fractions were excluded by centrifugation at 700×*g* for 5 min at 4 °C, and the postnuclear supernatant was then centrifuged at 100,000×*g* for 1 h at 4 °C. The resulting supernatant S100 was used as the cytosolic fraction, while the P100 pellet was resuspended in a buffer containing 50 mM Tris–HCl, pH 7.5, 150 mM NaCl, 1% Triton X-100, a cocktail of phosphatase and protease inhibitors without EDTA (Roche) and incubated on ice for 15 min. The mixture was subjected to a second centrifugation at 100,000 × *g* for 1 h at 4 °C. The resulting insoluble pellet (F-actin cytoskeleton fraction) (40) was resuspended in a buffer containing 50 mM Tris–HCl (pH 7.5), 150 mM NaCl, 1% Triton X-100, 2% SDS, and protease and phosphatase inhibitors) for WB or TEVP buffer (64) supplemented with 0.5% Triton-X-100 and a cocktail of protease inhibitors without EDTA (Roche) for F-actin-Alix coimmunoprecipitation. For the F-actin cytoskeleton fractions isolated in the presence of DTT, 0, 0.5, or 1.0 mM DTT in final concentration were included to PBS and three buffers aforementioned. No extra reducing agent was added before loading protein gels for Western blot analyses.

### Coimmunoprecipitation

Seventy micrograms F-actin cytoskeleton fraction proteins were incubated with 10 μg anti-F-actin antibody overnight at 4 °C while rotating. Samples were immunoprecipitated with PureProteome Protein A/G Mix Magnetic Beads for 1 h at RT. The beads were washed three times with TEVP buffer (64) supplemented with 0.5% Triton-X-100 and a cocktail of protease inhibitors without EDTA. Bound proteins were released by adding 1 × SDS-PAGE sample loading buffer to the beads and incubating at 70 °C for 10 min. Elutions were run on SDS polyacrylamide gels followed by immunoblotting with the indicated antibodies.

### Cytochalasin D treatment

Cytochalasin D (Sigma–Aldrich) was added to the culture medium for a final concentration of 10 μM and MEF cells were incubated for 1 h at 37 °C in 5% CO2. The cytochalasin D medium was replaced with fresh complete medium and cells were incubated for 0.5 h at 37 °C in 5% CO2 to recover F-actin cytoskeleton.

### Immunofluorescence and imaging

Culture cells were fixed in 4% paraformaldehyde in PBS at RT for 15 min and permeabilized with 0.1 % saponin in PBS and blocked for 1 h with 10% normal donkey serum in blocking buffer (PBS, 0.1 % bovine serum albumin, and 0.5% Tween 20). Fixed cells were immunostained with primary antibodies diluted in blocking buffer overnight at 4 °C. After three washings using blocking buffer, Cy3 donkey anti-rabbit IgG (Jackson Laboratories) was used as the secondary antibody and Alexa Fluor 488 dye conjugated phalloidin to probe F-actin (Invitrogen). Following three washings, cells were mounted with ProLong Gold antifade reagent with 4′,6-diamidino-2-phenylindole (Invitrogen) before confocal microscopy imaging. Images were acquired on a Nikon C2 confocal microscope using NIS Elements software (https://www.microscope.healthcare.nikon.com/products/software/nis-elements). A macro was developed in ImageJ (https://imagej.nih.gov/ij/download.html) to analyze the fluorescence intensity of F-actin in cells. A threshold for detection was set above the average background florescence to quantify the mean fluorescence intensity per field and batched to all images using the same parameters.

### Statistical analyses

Statistical analyses were performed using GraphPad Prism (GraphPad Software Inc). Quantitative data are presented as mean ± SD. For comparisons between two groups, Student’s *t* test (unpaired, two-tailed) was used. Groups were considered different significantly when *p* < 0.05. For all quantifications, at least three independent experiments were performed. Number of replicates is specified in the figure legends.

## Data availability

The mass spectrometry proteomics data have been deposited to the ProteomeXchange Consortium *via* the PRIDE (65) partner repository with the dataset identifier PXD031450.

## Supporting information

All other data are included in the article and the supporting information.

## Conflict of interest

The authors declare that they have no conflicts of interest with the contents of this article.

## Supporting information

Supplementary Figure 1 Uncropped WB blots from Figure 1. Labeling is consistent with Figure 1. *A*–*H*, markers (lane1) were loaded onto the same gel and visualized by ChemiDoc MP Imaging System (Bio-Rad) at a differently optimized exposure. The splice borders are marked by solid lines

Supplementary Figure 2 *A*, uncropped WB blots from Figure 2*A*. *B*, crude exosomes isolated from WT and *Alix*^*−/−*^ MEFs were subjected to sucrose density gradient ultracentrifugation and fractions were immunoblotted and probed with anti-Alix antibody. Dashed lined box denotes the exosomal fractions and dotted the membrane blebs and apoptotic bodies. *C*–*D*, sucrose density gradient of exosomes isolated from WT and *Alix*^*−/−*^ MEFs were subjected to WBs using antibodies against CD9 (*C*) and CD81 (*D*). *E*, uncropped WB blots from Figure 2*B*. *A*–*E*, markers (lane1) were loaded onto the same gel and visualized by ChemiDoc MP Imaging System (Bio-Rad) at a differently optimized exposure. The splice borders are marked by solid lines. MEF, mouse embryonic fibroblast

Supplementary Figure 3 *A*, uncropped WB blots from Figure 2*C*. *B*, D3 myocyte exosomes were subjected to sucrose density gradient and fractions were probed with anti-Alix antibody on WB. Exosomes from *Alix*^*−/−*^ primary cell culture were used as a negative control to validate specificity of Alix antibody. *C* and *D*, sucrose density gradient of exosomes isolated from WT and *Alix*^*−/−*^ D3 myocytes were subjected to WBs using antibodies against CD9 (*C*) and CD81 (*D*). *E*–*G*, uncropped WB blots from Figure 2, *D*–*F*. labeling is consistent with Figure 2. *A*–*G*, markers (lane1) were loaded onto the same gel and visualized by ChemiDoc MP Imaging System (Bio-Rad) at a differently optimized exposure. The splice borders are marked by solid lines

Supplementary Figure 4 *A* and *B*, uncropped WB blots from Figure 3*B*. Labeling is consistent with Figure 3*B*. *C*, Coomassie stained blot was used for loading. *D*, uncropped WB blot from Figure 3*C*. Labeling is consistent with Figure 3*C*. *E*, Superdex 200 increase 10/300 GL size-exclusion chromatography markers. *F* and *G*, uncropped WB blots from Figure 3, *F* and *G*. Labeling is consistent with Figure 3, *F* and *G*. *A*–*C*, *F*, and *G*, markers (lane1) were loaded onto the same gel and visualized by ChemiDoc MP Imaging System (Bio-Rad) at a differently optimized exposure. The splice borders are marked by solid lines

Supplementary Figure 5 Uncropped WB blots from Figure 4, *A*–*C*. Labeling is consistent with Figure 4. *B*, *C*, and *E*–*N*, markers (lane1) were loaded onto the same gel and visualized by ChemiDoc MP Imaging System (Bio-Rad) at a differently optimized exposure. The splice borders are marked by solid lines

Supplementary Figure 6 *A*–*C*, quantification of WBs corresponding to Figure 4, *A*–*C*. *D* and *E*, sucrose density gradient of exosomes from WT and *Alix*^*−/−*^ MEFs (*D*) and D3 myocytes (*E*) were subjected to WBs using antibodies against syntenin. *F*, quantification of WBs corresponding to Figure 4*E*. *G*–*L*, uncropped WB blots from Figure 4*E*. Labeling is consistent with Figure 4*E*. *D*, *E*, and *G*–*K*, markers (lane1) were loaded onto the same gel and visualized by ChemiDoc MP Imaging System (Bio-Rad) at a differently optimized exposure. The splice borders are marked by solid lines. MEF, mouse embryonic fibroblast

Supplementary Figure 7 *A*, RT-qPCR of *Alix* and *syntenin* in WT and *Alix*^*−/−*^ MEFs (*n* = 4). mRNA levels are normalized by 1*8S rRNA* and relative to WT control. Values are expressed as means ± SD. Statistical analysis was performed using Student *t* test; ∗∗∗∗*P*<0.0001. *B* and *C*, quantification of WBs corresponding to Figure 4*F* and *G*. *D*–*O*, uncropped WB blots from Figure 4, *F* and *G*,. Labeling is consistent with Figures 4, *F* and *G*. *D*–*H* and *J*–*N*, markers (lane1) were loaded onto the same gel and visualized by ChemiDoc MP Imaging System (Bio-Rad) at a differently optimized exposure. The splice borders are marked by solid lines. RT-qPCR, real-time quantitative PCR

Supplementary Figure 8 Uncropped WB blots from Figure 5, *A* and *B*. Labeling is consistent with Figure 5, *A* and *B*. *A*, *B*, *D*, and *E*, markers (lane1) were loaded onto the same gel and visualized by ChemiDoc MP Imaging System (Bio-Rad) at a differently optimized exposure. The splice borders are marked by solid lines

Supplementary Figure 9 Uncropped WB blots from Figure 6, *B*–*D*. Labeling is consistent with Figure 6, *B*–*D*. *A*, *D*, and *E*, markers (lane1) were loaded onto the same gel and visualized by ChemiDoc MP Imaging System (Bio-Rad) at a differently optimized exposure. The splice borders are marked by solid lines

Supplementary Figure 10 *A*–*C*, uncropped WB blots from Figure 7, *A*–*C*. Labeling is consistent with Figure 7, *A*–*C*. *D*, *Alix*^*−/−*^ MEFs transduced with MSCV expressing WT Alix or Alix harboring C814S mutant (C814S). Cells were treated with 10 μM cytochalasin D (CD) for 1 h before immunofluorescent staining with anti-Alix antibody (*red*) and Alexa Flour 488 dye conjugated phalloidin to probe F-actin (*green*). The scale bars represent 100 μm. *A*–*C*, markers (lane1) were loaded onto the same gel and visualized by ChemiDoc MP Imaging System (Bio-Rad) at a differently optimized exposure. The splice borders are marked by solid lines. MEF, mouse embryonic fibroblast; MSCV, Murine stem cell virus
