# Supplementary figures and images for: Distinct functions of dimeric and monomeric scaffold protein Alix in regulating F-actin assembly and loading of exosomal cargo
Source: J Biol Chem. 2022 Aug 27;298(10):102425. doi: 10.1016/j.jbc.2022.102425 (PMC9531180; doi:10.1016/j.jbc.2022.102425)

Suppl. Figure 1

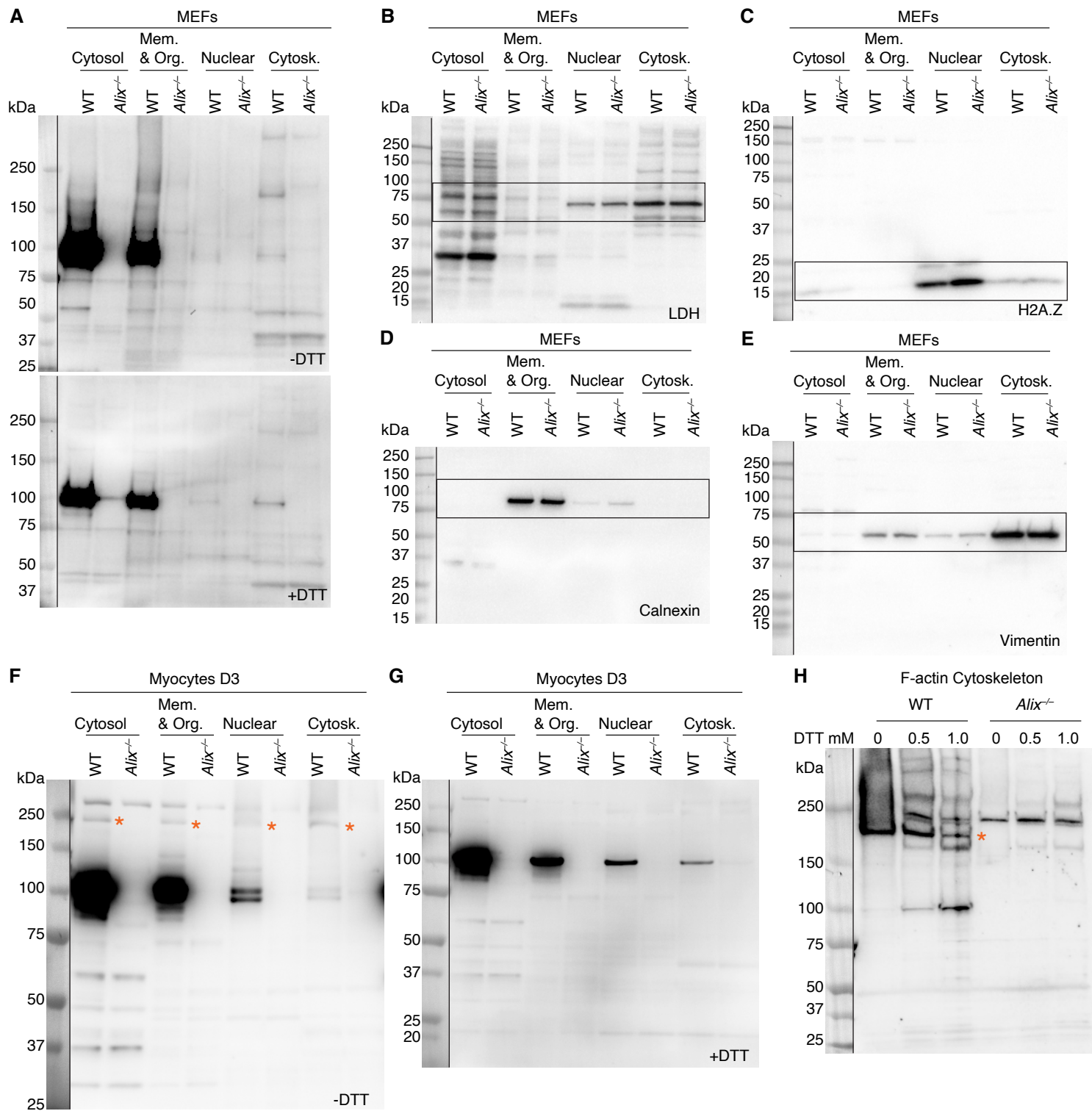

Supplement: Supplementary Figure 1 — Uncropped WB blots from Figure 1. Labeling is consistent with Figure 1. A–H, markers (lane1) were loaded onto the same gel and visualized by ChemiDoc MP Imaging System (Bio-Rad) at a differently optimized exposure. The splice borders are marked by solid lines [file mmc1.pdf]

Suppl. Figure 2

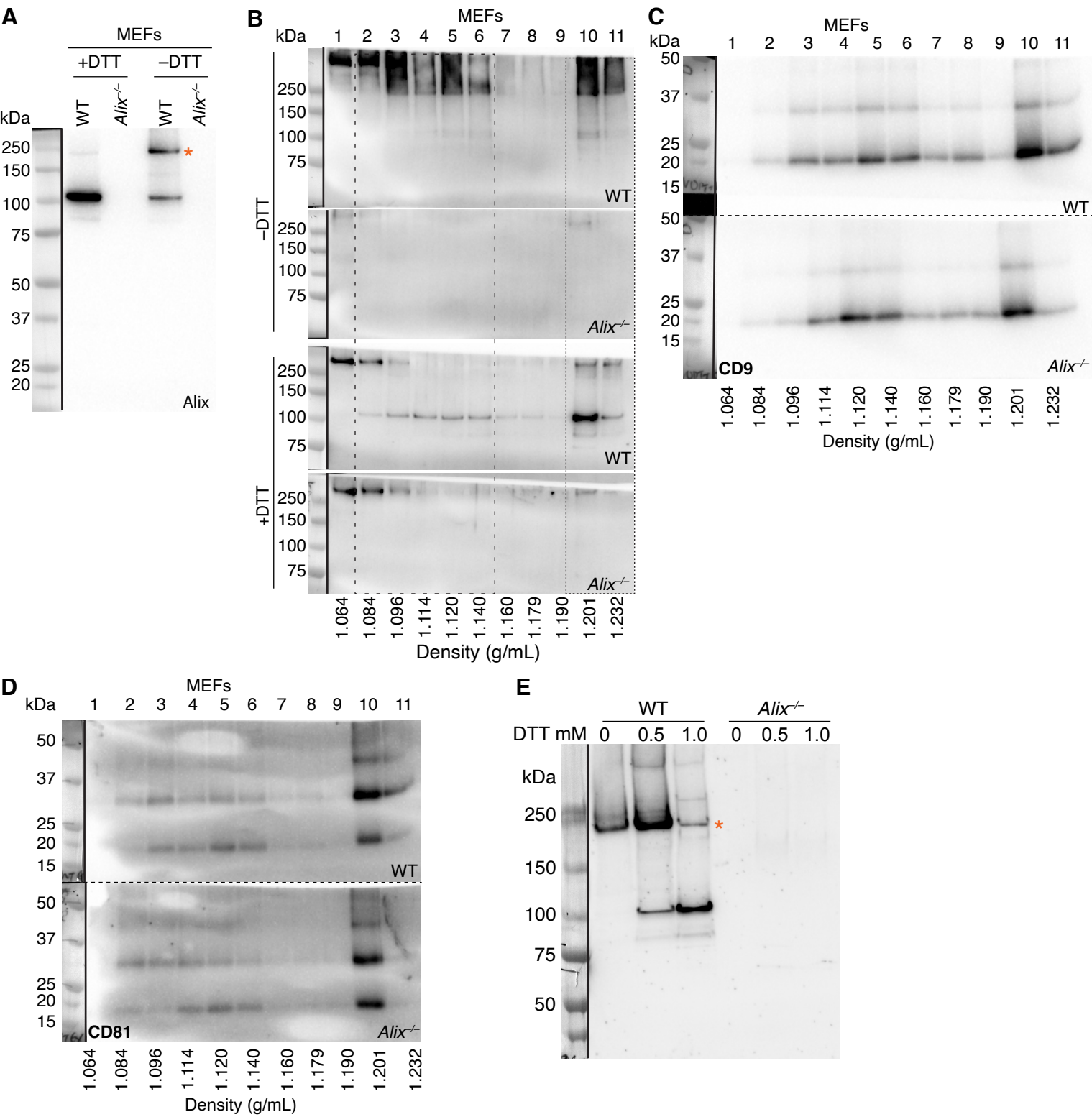

Supplement: Supplementary Figure 2 — A, uncropped WB blots from Figure 2A. B, crude exosomes isolated from WT and Alix−/− MEFs were subjected to sucrose density gradient ultracentrifugation and fractions were immunoblotted and probed with anti-Alix antibody. Dashed lined box denotes the exosomal fractions and dotted the membrane blebs and apoptotic bodies. C–D, sucrose density gradient of exosomes isolated from WT and Alix−/− MEFs were subjected to WBs using antibodies against CD9 (C) and CD81 (D). E, uncropped WB blots from Figure 2B. A–E, markers (lane1) were loaded onto the same gel and visualized by ChemiDoc MP Imaging System (Bio-Rad) at a differently optimized exposure. The splice borders are marked by solid lines. MEF, mouse embryonic fibroblast [file mmc2.pdf]

Suppl. Figure 4

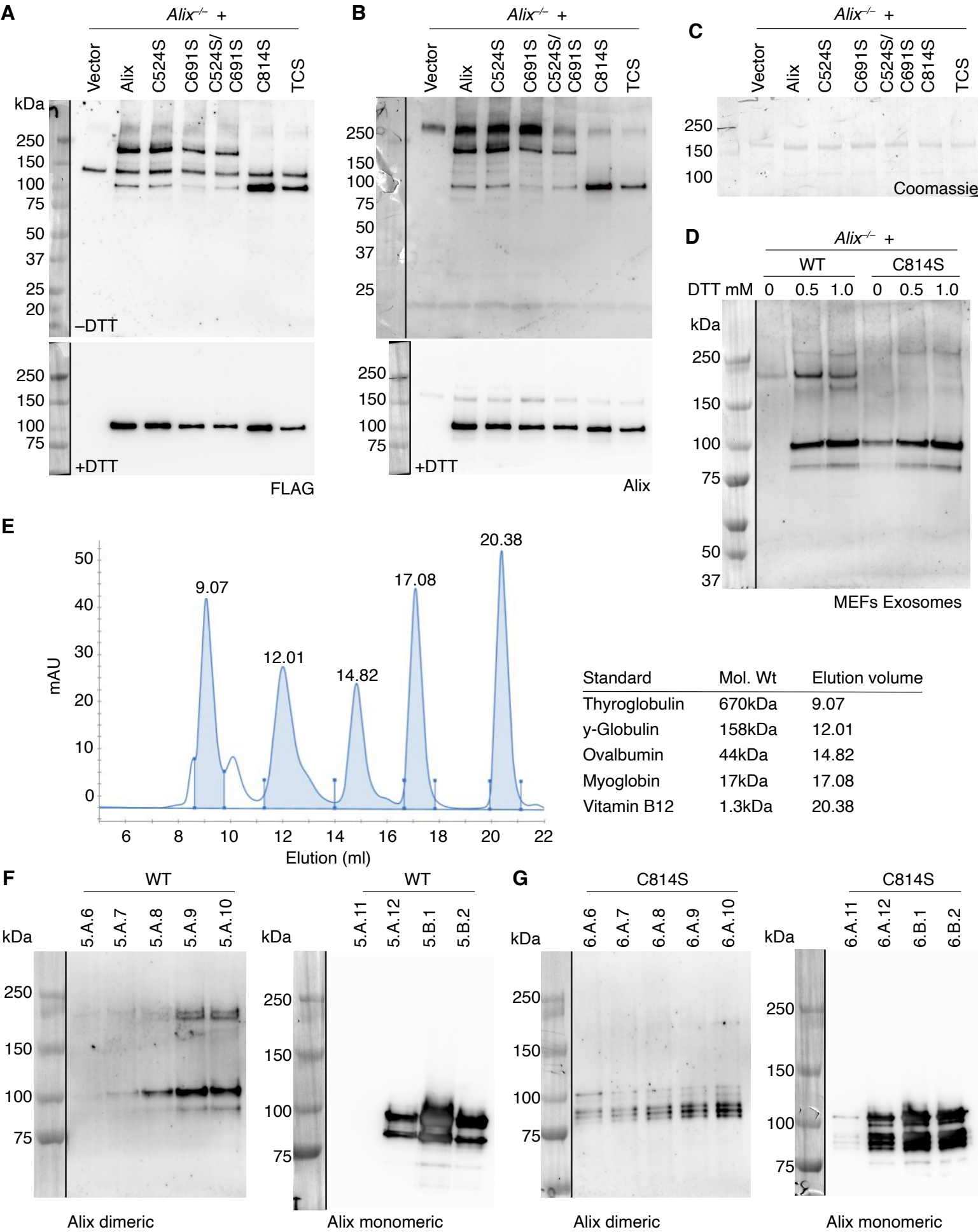

Supplement: Supplementary Figure 4 — A and B, uncropped WB blots from Figure 3B. Labeling is consistent with Figure 3B. C, Coomassie stained blot was used for loading. D, uncropped WB blot from Figure 3C. Labeling is consistent with Figure 3C. E, Superdex 200 increase 10/300 GL size-exclusion chromatography markers. F and G, uncropped WB blots from Figure 3, F and G. Labeling is consistent with Figure 3, F and G. A–C, F, and G, markers (lane1) were loaded onto the same gel and visualized by ChemiDoc MP Imaging System (Bio-Rad) at a differently optimized exposure. The splice borders are marked by solid lines [file mmc4.pdf]

Suppl. Figure 5

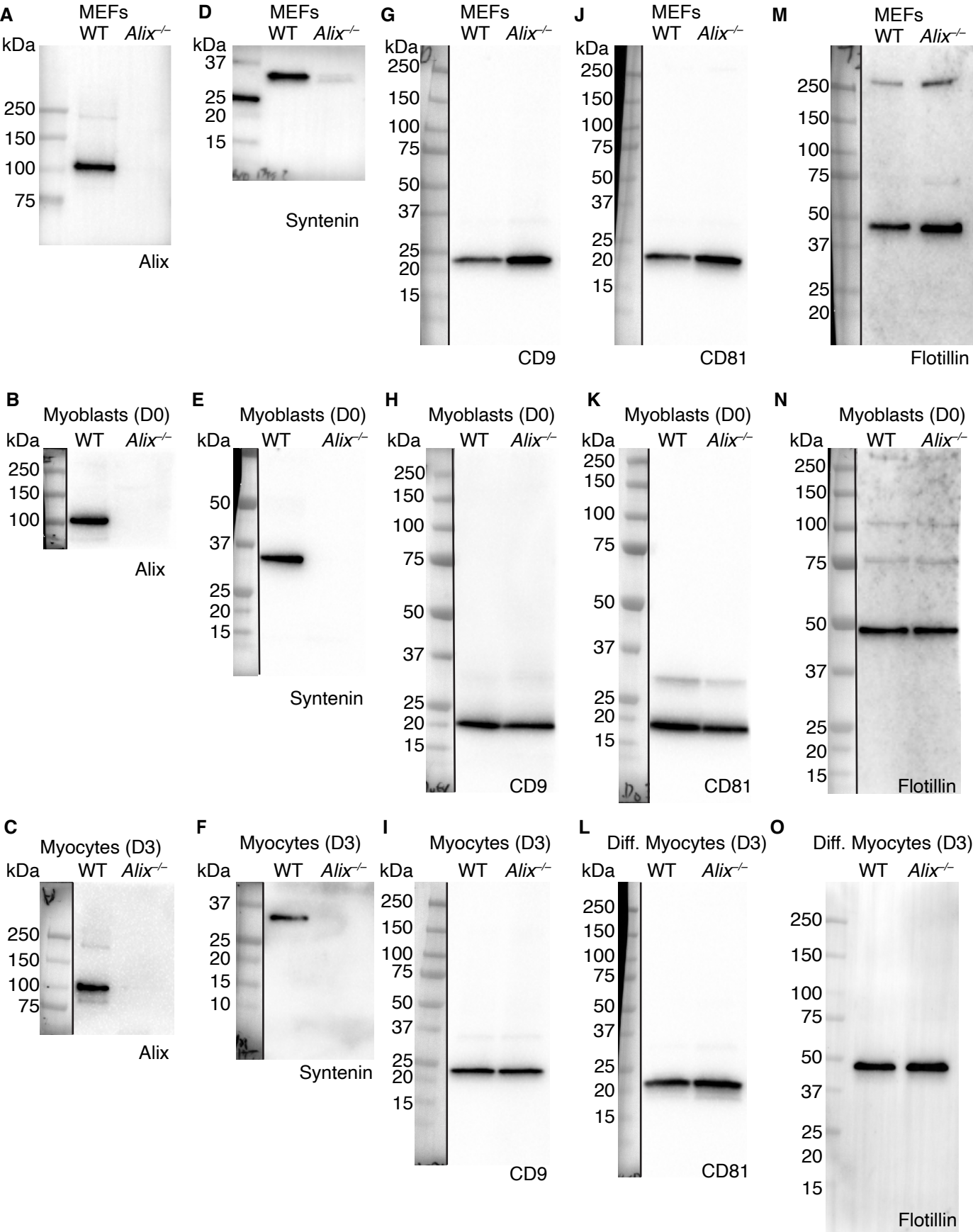

Supplement: Supplementary Figure 5 — Uncropped WB blots from Figure 4, A–C. Labeling is consistent with Figure 4. B, C, and E–N, markers (lane1) were loaded onto the same gel and visualized by ChemiDoc MP Imaging System (Bio-Rad) at a differently optimized exposure. The splice borders are marked by solid lines [file mmc5.pdf]

Suppl Fig 6

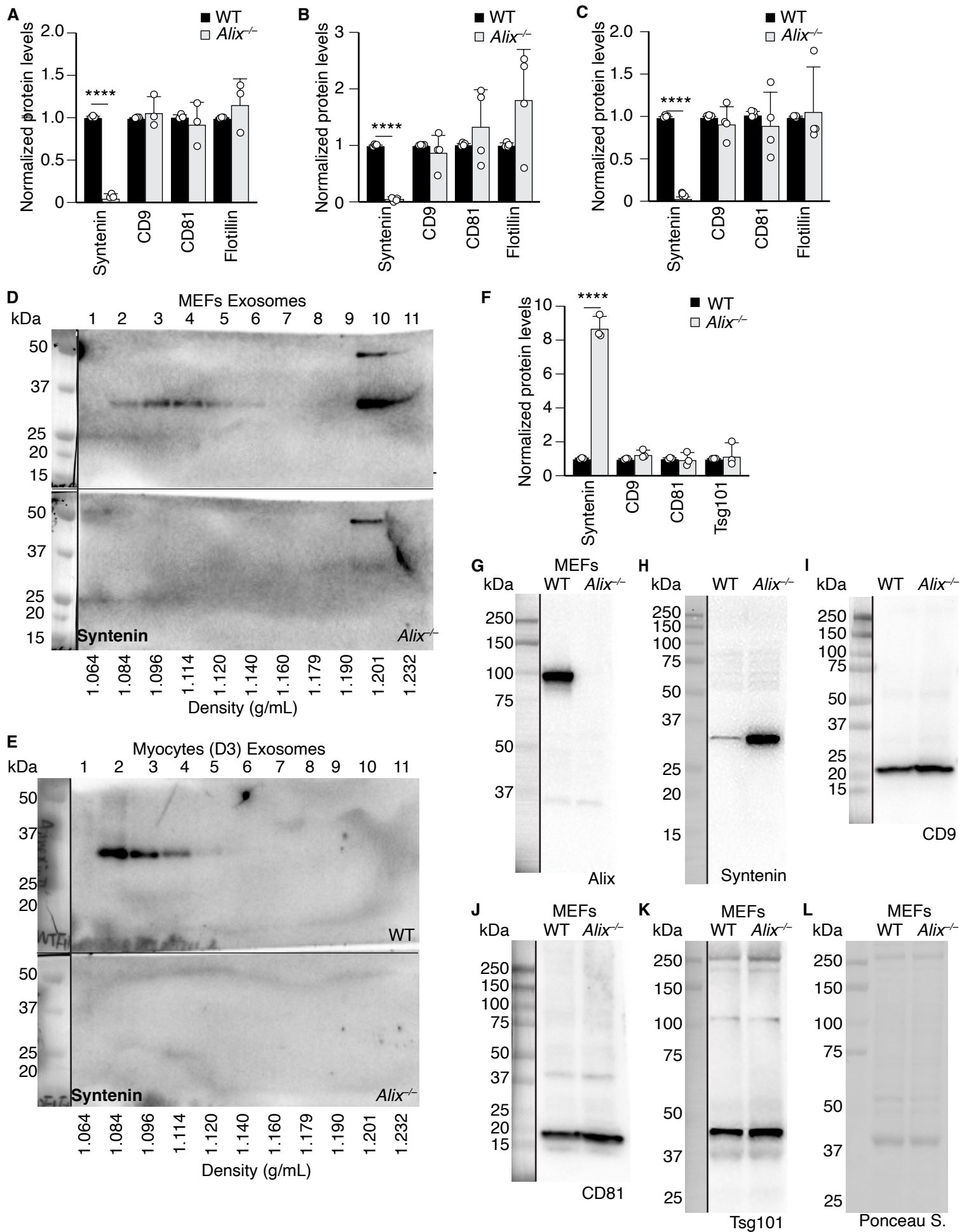

Supplement: Supplementary Figure 6 — A–C, quantification of WBs corresponding to Figure 4, A–C. D and E, sucrose density gradient of exosomes from WT and Alix−/− MEFs (D) and D3 myocytes (E) were subjected to WBs using antibodies against syntenin. F, quantification of WBs corresponding to Figure 4E. G–L, uncropped WB blots from Figure 4E. Labeling is consistent with Figure 4E. D, E, and G–K, markers (lane1) were loaded onto the same gel and visualized by ChemiDoc MP Imaging System (Bio-Rad) at a differently optimized exposure. The splice borders are marked by solid lines. MEF, mouse embryonic fibroblast [file mmc6.pdf]

Suppl. Figure 7

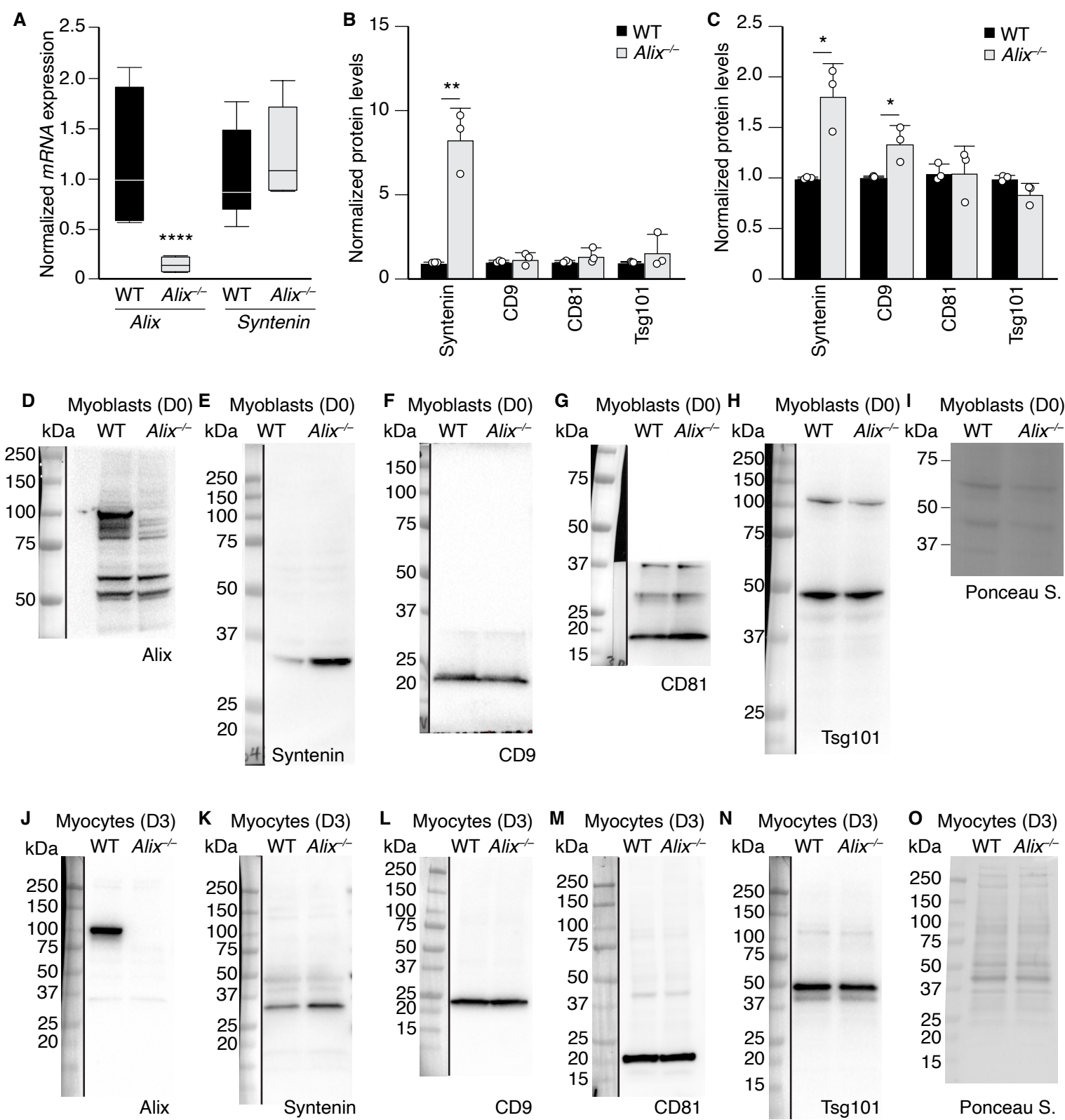

Supplement: Supplementary Figure 7 — A, RT-qPCR of Alix and syntenin in WT and Alix−/− MEFs (n = 4). mRNA levels are normalized by 18S rRNA and relative to WT control. Values are expressed as means ± SD. Statistical analysis was performed using Student t test; ∗∗∗∗P<0.0001. B and C, quantification of WBs corresponding to Figure 4F and G. D–O, uncropped WB blots from Figure 4, F and G,. Labeling is consistent with Figures 4, F and G. D–H and J–N, markers (lane1) were loaded onto the same gel and visualized by ChemiDoc MP Imaging System (Bio-Rad) at a differently optimized exposure. The splice borders are marked by solid lines. RT-qPCR, real-time quantitative PCR [file mmc7.pdf]

Suppl. Figure 8

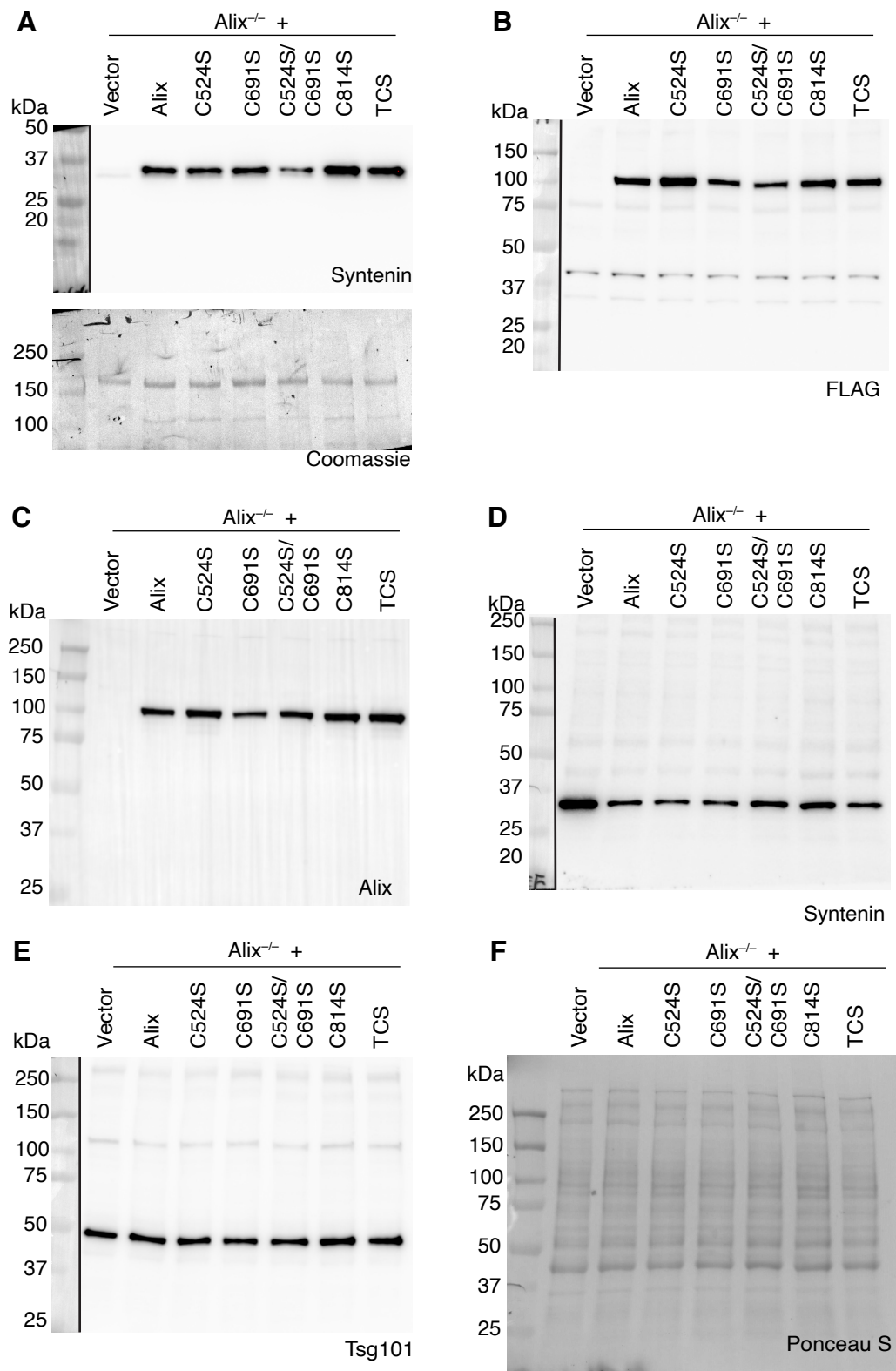

Supplement: Supplementary Figure 8 — Uncropped WB blots from Figure 5, A and B. Labeling is consistent with Figure 5, A and B. A, B, D, and E, markers (lane1) were loaded onto the same gel and visualized by ChemiDoc MP Imaging System (Bio-Rad) at a differently optimized exposure. The splice borders are marked by solid lines [file mmc8.pdf]

Supple Figure 9

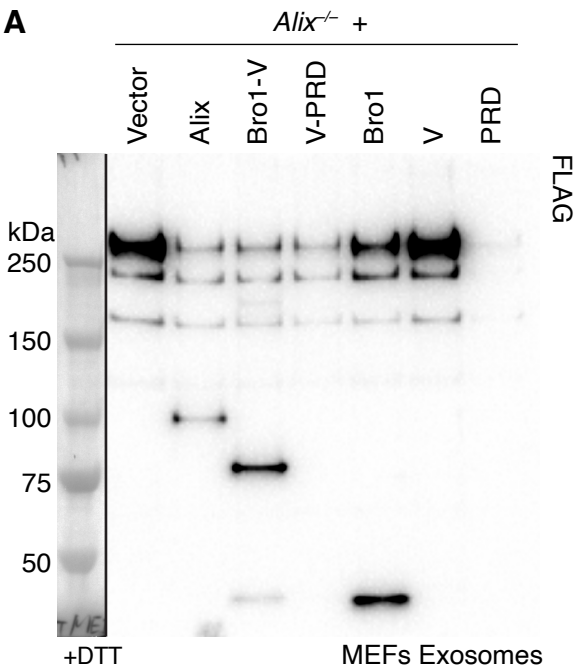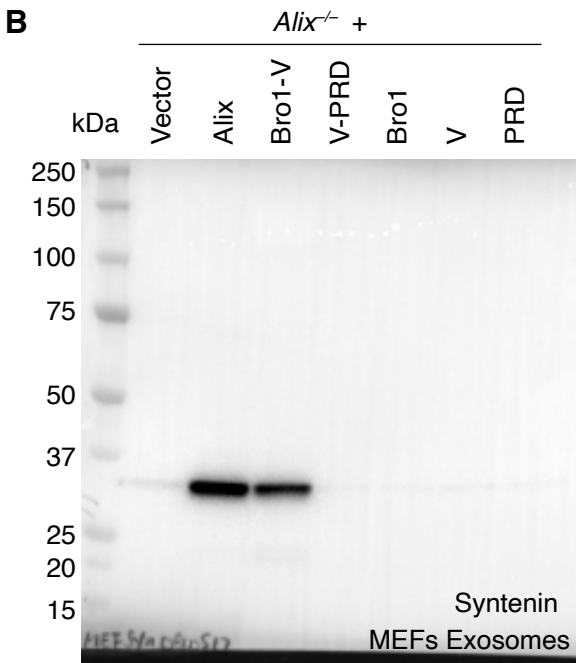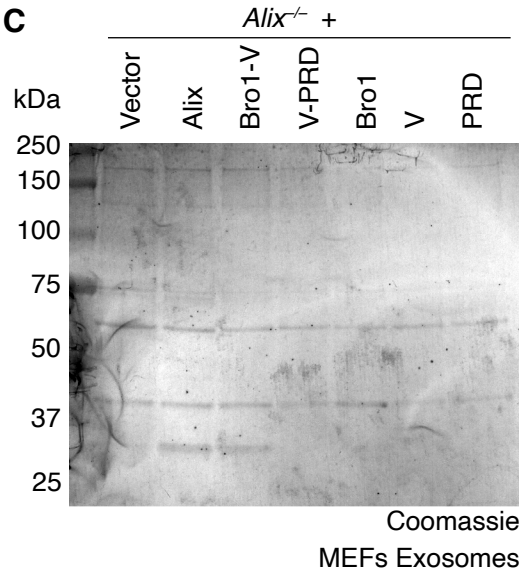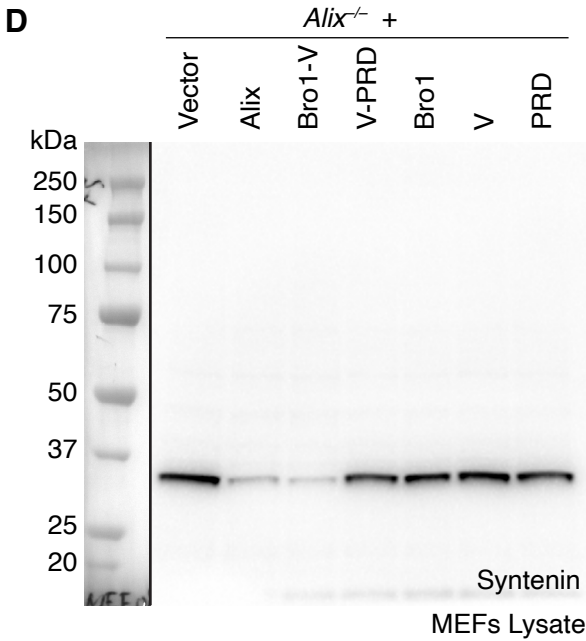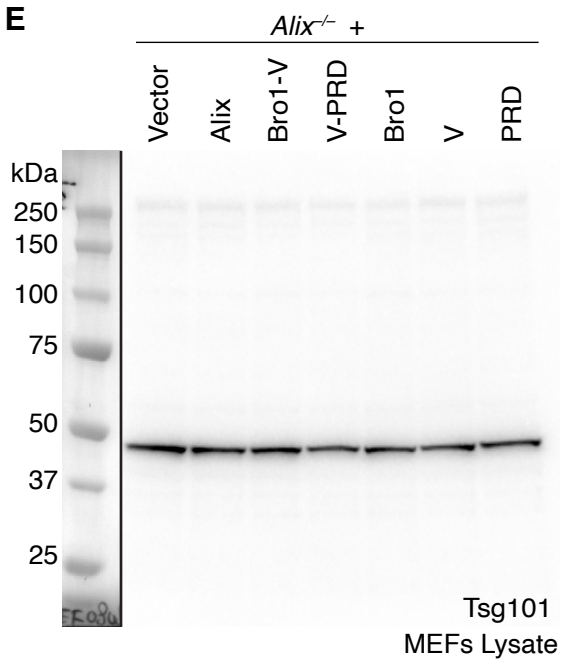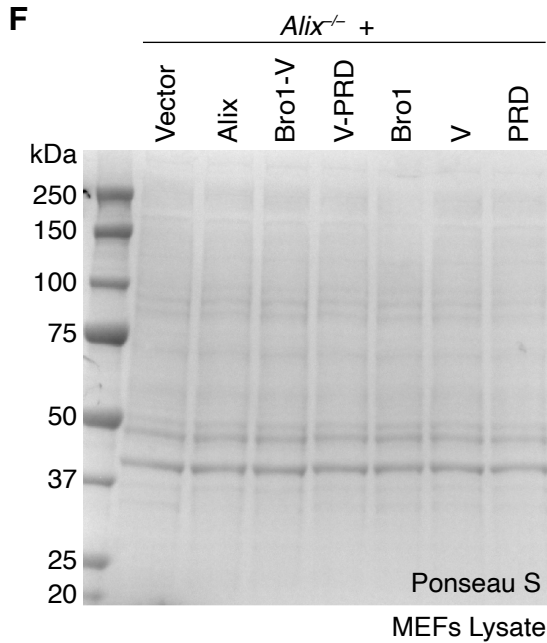

Supplement: Supplementary Figure 9 — Uncropped WB blots from Figure 6, B–D. Labeling is consistent with Figure 6, B–D. A, D, and E, markers (lane1) were loaded onto the same gel and visualized by ChemiDoc MP Imaging System (Bio-Rad) at a differently optimized exposure. The splice borders are marked by solid lines [file mmc9.pdf]

Suppl Figure 10

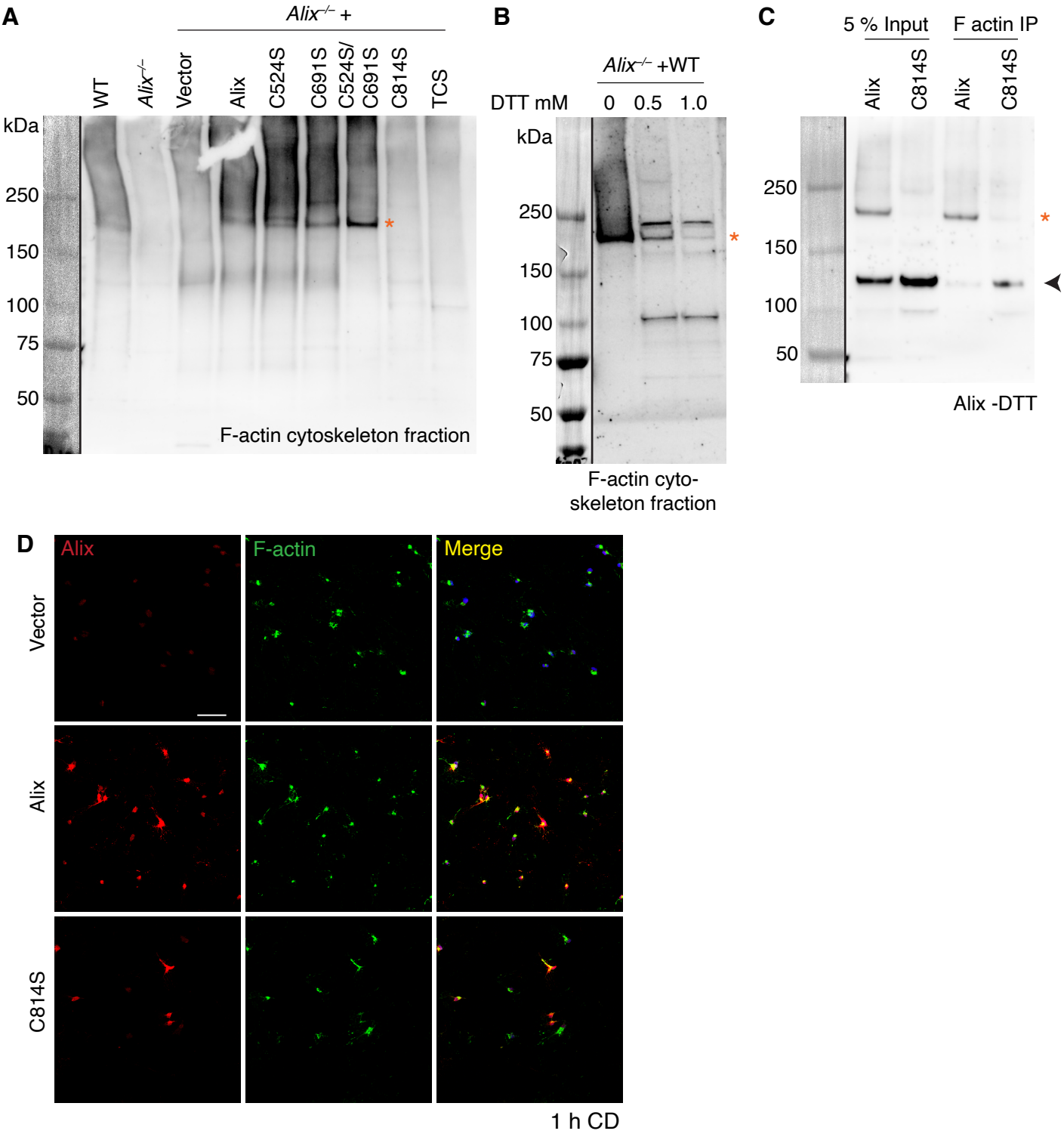

Supplement: Supplementary Figure 10 — A–C, uncropped WB blots from Figure 7, A–C. Labeling is consistent with Figure 7, A–C. D, Alix−/− MEFs transduced with MSCV expressing WT Alix or Alix harboring C814S mutant (C814S). Cells were treated with 10 μM cytochalasin D (CD) for 1 h before immunofluorescent staining with anti-Alix antibody (red) and Alexa Flour 488 dye conjugated phalloidin to probe F-actin (green). The scale bars represent 100 μm. A–C, markers (lane1) were loaded onto the same gel and visualized by ChemiDoc MP Imaging System (Bio-Rad) at a differently optimized exposure. The splice borders are marked by solid lines. MEF, mouse embryonic fibroblast; MSCV, Murine stem cell virus [file mmc10.pdf]
