# Supplementary material for: Distinct functions of dimeric and monomeric scaffold protein Alix in regulating F-actin assembly and loading of exosomal cargo
Source: J Biol Chem. 2022 Aug 27;298(10):102425. doi: 10.1016/j.jbc.2022.102425 (PMC9531180; doi:10.1016/j.jbc.2022.102425)

Suppl. Figure 3

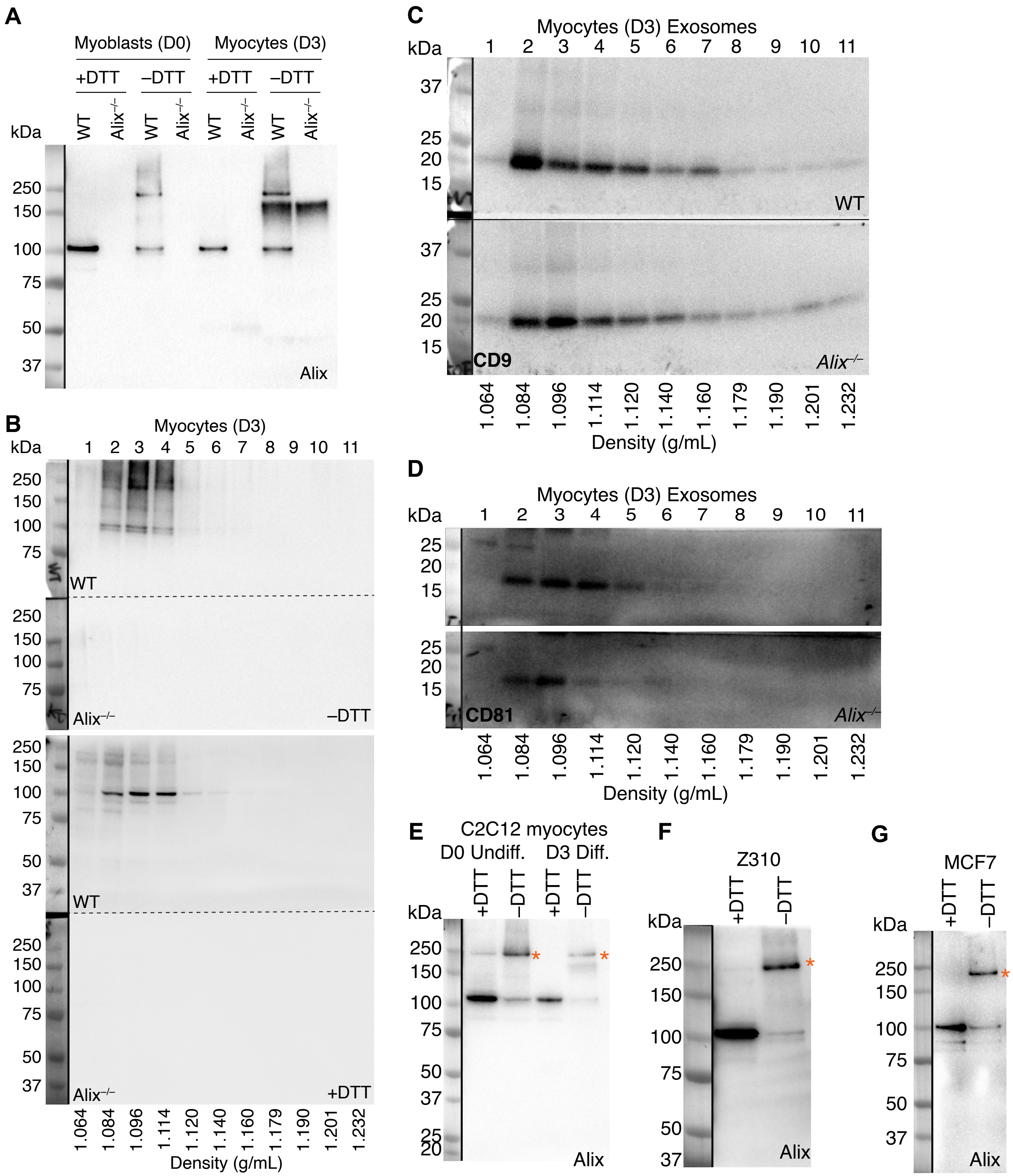

Supplement: Supplementary Figure 3 — A, uncropped WB blots from Figure 2C. B, D3 myocyte exosomes were subjected to sucrose density gradient and fractions were probed with anti-Alix antibody on WB. Exosomes from Alix−/− primary cell culture were used as a negative control to validate specificity of Alix antibody. C and D, sucrose density gradient of exosomes isolated from WT and Alix−/− D3 myocytes were subjected to WBs using antibodies against CD9 (C) and CD81 (D). E–G, uncropped WB blots from Figure 2, D–F. labeling is consistent with Figure 2. A–G, markers (lane1) were loaded onto the same gel and visualized by ChemiDoc MP Imaging System (Bio-Rad) at a differently optimized exposure. The splice borders are marked by solid lines [file mmc3.pdf]
