# Supplementary material for: Distinct functions of dimeric and monomeric scaffold protein Alix in regulating F-actin assembly and loading of exosomal cargo
Source: J Biol Chem. 2022 Aug 27;298(10):102425. doi: 10.1016/j.jbc.2022.102425 (PMC9531180; doi:10.1016/j.jbc.2022.102425)
Supplement: Table S1 [file mmc11.pdf]

Table S1 Comparative proteomics analysis of Alix -/- and WT exosomes

Fibroblast

Protein name	Gene	Uniques peptides		Mol. Weigh	Score	Ms/MS count		LFG intensity Exp		*Alix* $^{-/-}$ /WT
		Alix $^{-/-}$	WT	(kDa)		*Alix* $^{-/-}$	WT	*Alix* $^{-/-}$	WT	LFQ
Alix	Alix	0	37	96.023	166.05	0	67	0	1.80E+08	0.00
Syntenin	Sdcbp	3	15	32.251	88.77	5	38	4.74E+06	2.14E+08	0.02
CD9 antigen	Cd9	3	5	25.258	323.31	9	13	9.84E+07	7.71E+07	1.28
CD81 antigen	Cd81	3	3	25.814	71.82	7	12	5.18E+07	5.05E+07	1.03
Tumor susceptibility gene 101 protein	Tsg101	5	5	44.123	19.21	7	6	1.39E+07	1.09E+07	1.28
Tetraspanin;CD63 antigen	Cd63	1	1	25.766	3.38	2	1	9.21E+06	1.35E+07	0.68

D0

Protein name	Gene	Uniques peptides		Mol. Weigh	Score	Ms/MS count		LFG intensity Exp		*Alix* $^{-/-}$ /WT
		Alix $^{-/-}$	WT	(kDa)		*Alix* $^{-/-}$	WT	*Alix* $^{-/-}$	WT	LFQ
Alix	Alix	0	46	96.023	223.60	0	102	0	5.55E+08	0.00
Syntenin	Sdcbp	6	23	32.251	237.41	7	58	2.75E+07	6.14E+08	0.04
CD9 antigen	Cd9	4	2	25.258	179.71	6	6	4.46E+07	1.85E+07	2.41
CD81 antigen	Cd81	5	7	25.814	172.34	19	25	3.66E+08	4.23E+08	0.87
Tumor susceptibility gene 101 protein	Tsg101	7	8	44.123	71.87	10	11	6.75E+07	5.00E+07	1.35
Tetraspanin;CD63 antigen	Cd63	2	1	25.766	23.99	2	1	8.65E+07	5.97E+07	1.45

D3

Protein name	Gene	Uniques peptides		Mol. Weigh	Score	Ms/MS count		LFG intensity Exp		*Alix* $^{-/-}$ /WT
		Alix $^{-/-}$	WT	(kDa)		*Alix* $^{-/-}$	WT	*Alix* $^{-/-}$	WT	LFQ
Alix	Alix	0	23	96.023	169.94	0	35	0	4.42E+08	0.00
Syntenin	Sdcbp	0	10	32.251	60.70	0	19	0.00E+00	4.35E+08	0.00
CD9 antigen	Cd9	4	3	25.258	198.01	7	6	2.79E+08	2.78E+08	1.00
CD81 antigen	Cd81	3	4	25.814	165.48	20	17	9.59E+08	1.33E+09	0.72
Tumor susceptibility gene 101 protein	Tsg101	7	4	44.123	32.40	10	6	5.57E+07	6.92E+07	0.80
Tetraspanin;CD63 antigen	Cd63	2	2	25.766	4.22	3	4	2.43E+07	6.47E+07	0.38
